# Optimized cumate toolkit for tunable protein expression during *in vitro* and *in vivo* studies of *Burkholderia cenocepacia*

**DOI:** 10.1128/aem.00532-26

**Published:** 2026-06-09

**Authors:** Hamza Tahir, Kristian I. Karlic, Godfrey Mwiti, Nichollas E. Scott

**Affiliations:** 1 Department of Microbiology and Immunology, University of Melbourne, Peter Doherty Institute for Infection and Immunity, Melbourne, Victoria, Australia; Washington University in St Louis, St. Louis, Missouri, USA

**Keywords:** glycosylation, *Burkholderia cenocepacia*, *Burkholderia*, cumate induction, proteomics, glycoproteomics

## Abstract

Inducible gene expression is pivotal to dissect bacterial physiology and virulence mechanisms. Across the *Burkholderia* genus, a limited range of inducible systems currently exist that allow tight regulation. In this study, we engineer a set of cumate inducible vectors for use in *Burkholderia cenocepacia* that offer minimal basal expression and the ability to control *B. cenocepacia* gene expression in eukaryotic cells. Through mutagenesis-based studies of cumate circuits and the cumate regulator (CymR), we have generated an optimized cumate circuit (P_CymRC_/CymR_GV_) which allows the tight and tunable control of protein expression in *B. cenocepacia,* as assessed by fluorescent and protein O-linked glycosylation analysis. Using comparative proteomics, we find that cumate elicits discrete alterations within the *B. cenocepacia* proteome that are orthogonal to those seen in widely used rhamnose-based induction systems, supporting cumate induction as a complementary approach for protein expression in *Burkholderia* spp. Leveraging the cell permeability of cumate and the generation of CTX-based chromosomal integration vectors, we show that inducible control of protein expression is achievable during intracellular replication of *B. cenocepacia*. By controlling intracellular expression of O-linked protein glycosylation, we demonstrate that protein glycosylation contributes to optimal intracellular replication of *B. cenocepacia*. This work demonstrates that cumate inducible systems allow precise and tunable gene expression in *Burkholderia* even within a host-pathogen context.

**IMPORTANCE:**

This work establishes optimized cumate-inducible vectors for use in *Burkholderia cenocepacia*, addressing the need for alternative inducers to available carbohydrate systems. We show that cumate-inducible vectors allow precise control of gene expression even within eukaryotic cells, providing a new and orthogonal way to temporally control protein induction. Utilizing cumate-based induction, we demonstrate the importance of O-linked protein glycosylation for optimal intracellular replication in *B. cenocepacia*, highlighting the potential of cumate systems to explore host-pathogen interactions. Combined, this work shows cumate-inducible vectors extend the range of studies which can be undertaken to dissect *B. cenocepacia* physiology and virulence.

## INTRODUCTION

Precise, titratable modulation of gene regulation is indispensable to study bacterial physiology and virulence at both mechanistic and systems levels (1–3). Ideally, inducible systems should provide rapid response kinetics, high dynamic range, and minimal basal expression to enable the precise assessment of gene functions in a controllable manner (4–6). Over the past 50 years, a diverse array of inducible systems responsive to naturally occurring and synthetic or non-metabolizable inducible agents have been developed (5, 7–10). In commonly utilized inducible systems, the control of gene expression is typically mediated by regulatory circuits composed of promoters and repressor pairs with well-known examples including P_lac_/LacI (11), P_tet_/TetR (12), P_BAD_/AraC, and P_rha_/RhaRS (13) systems. To improve the control of gene expression, synthetic biology, including protein engineering and rational DNA regulatory designs, is increasingly utilized to generate refined regulatory circuits (3, 9, 10). To date, several engineered regulatory circuits have been reported with a key goal of these systems being to reduce basal expression and enhance specificity of circuits compared to the canonical/natural regulatory systems (3, 14, 15). While early studies focused on well-known regulatory circuits to achieve these goals (16), recent efforts have sought to expand the toolbox of engineered regulatory systems by refining novel circuits for diverse applications, such as metabolic engineering (17) with one such regulatory circuit being the 4-isopropylbenzoate inducible circuit.

4-Isopropylbenzoate, herein referred to as cumate, is the native ligand for the P_Cym_/CymR inducible circuit identified within the *cmt* operon of *Pseudomonas putida* F1 (18–20). In this system, repression of cumate catabolism is mediated by the binding of CymR to a Cumate Operator (CuO) DNA sequence, which in the presence of cumate dissociates, allowing gene expression downstream of CuO (20). Since cumate is cell permeable, nontoxic, and non-metabolizable in the absence of a cumate metabolism pathway (19, 20), several teams have used the canonical P_Cym_/CymR inducible circuit for titratable gene expression in *Escherichia coli* (21, 22), *Pseudomonas aeruginosa* (23), as well as non-model organisms such as *Streptomyces* spp. (24) and members of the *Alphaproteobacteria* (25). The high permeability and non-toxic nature of cumate make this inducer an attractive option in systems where other agents may be poorly tolerated or excluded, which is best exemplified by its use in mammalian systems, where eukaryotic cumate-inducible circuits have been constructed (26). In 2018, Meyer et al. engineered a synthetic cumate circuit, referred to here as P_CymRC_/CymR_AM_, suggested to possess reduced basal expression and minimal antagonism with other commonly used inducers (3). Due to the accessibility of this engineered variant, several teams have incorporated P_CymRC_/CymR_AM_ in plasmids designed for non-model organisms and demonstrated cumate induction of fluorescent reporters (27, 28). However, as this engineered regulatory circuit has only been evaluated in a limited context to date, it remains unclear if its reported characteristics are a true reflection of its performance, especially in non-model organisms such as members of the *Burkholderia* genera.

*Burkholderia* species are ubiquitous environmental organisms, and while the majority are non-pathogenic, members of *the Burkholderia cepacia* and *Burkholderia pseudomallei* complexes are associated with serious human infections (29–31). In the *Burkholderia cepacia* complex, *Burkholderia cenocepacia* is a significant opportunistic pathogen in patients with cystic fibrosis (CF), where it infects and persists intracellularly within host cells, driving a progressive decline in lung function (31, 32). To date, the study of *B. cenocepacia* pathogenesis has been impeded by both the innate multidrug resistance of isolates, which limits the resistance markers available for creating gene knockouts (33, 34), as well as a paucity of tools for precise complementation and/or protein overexpression studies. In *B. cenocepacia*, inducible protein expression studies have primarily utilized two carbohydrate-based inducer systems, P_BAD_/AraC (35) and P_rha_/RhaRS (36), activated by arabinose and rhamnose, respectively. While these circuits are functional in a range of *Burkholderia* species, P_BAD_/AraC is known to display basal expression (37) and requires high concentrations of L-arabinose (2%–3%) for the induction of uniform protein expression (36, 38). In contrast, P_rha_/RhaRS provides tight and uniform regulation of gene expression (36), making it the preferred induction system and leading to its use in several recently developed CRISPR systems (39, 40) as well as the assessment of essential genes (41, 42). However, we have previously observed that the induction of the P_rha_/RhaRS-based system with even modest rhamnose levels (0.1%) can evoke large proteomic changes in *B. cenocepacia* (43). While teams have made recent improvements to the dynamic range of P_rha_/RhaRS for the control of essential genes in *B. cenocepacia* (42), orthogonal chemically inert inducers would be highly beneficial for the control of protein expression in *Burkholderia* species.

In this work, we developed a set of cumate-inducible vectors to allow the tight regulation of protein expression in *B. cenocepacia*. Comparing available cumate regulatory circuits, using both fluorescent and an enzymatic reporter system to assess protein O-linked glycosylation, we demonstrate that currently available circuits show high basal expression, which limits their utility for the study of enzymatic processes. Using targeted mutagenesis, we show that the previously reported point mutations suggested to enhance CymR_AM_ performance increases basal expression and potentiates the responsiveness to cumate. By combining elements of the canonical and engineered CymR_AM_ cumate circuit, we have generated a new variant, P_CymRC_/CymR_GV_ that markedly reduces basal expression while preserving robust, dose-dependent induction. When deployed to control protein glycosylation, P_CymRC_/CymR_GV_ demonstrates precise, inducer-dependent modulation, with quantitative proteomics revealing distinct and orthogonal perturbations following cumate induction to the widely used rhamnose-induction system. Finally, we show that cumate-based induction provides a novel means to control protein expression within infection contexts. Collectively, this study establishes a novel set of cumate-inducible vectors for *B. cenocepacia,* and other *Burkholderia* species, that are orthogonal to current systems and are suitable to control protein expression within *in vivo* studies.

## RESULTS

### The cumate circuit P_Cym_/CymR is functional yet displays high basal expression in *B. cenocepacia*

**Fig 1:**
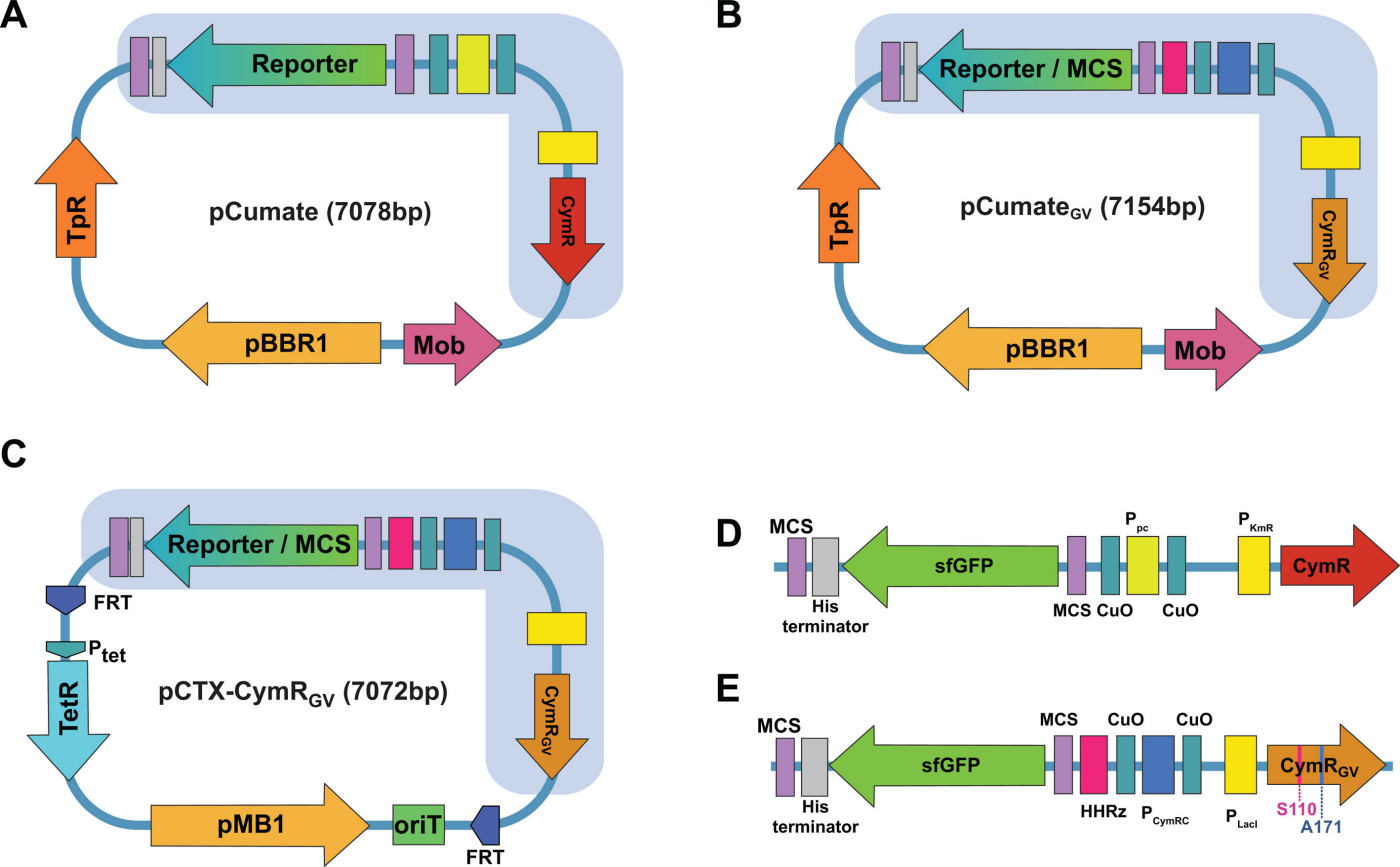
Schematic representation of cumate-inducible vectors and cumate circuit designs. (**A**) Plasmid pCumate containing the P_pc_/CymR regulatory circuit in a pBBR1 backbone containing a Trimethoprim resistance (Tp^R^) cassette, mobilization (Mob) elements, as well as regulatory and reporter modules contained within the shaded region denoted in panel D. (**B**) The optimized expression construct pCumate_GV_, incorporating CymR_GV_ (G110S, V171A) for enhanced regulation and reduced basal expression with the regulatory and reporter modules contained within the shaded region denoted in panel E. (**C**) The CTX-pCymR_GV_ integrative vector utilizing a Mini-CTX1 pMB1 backbone containing FRT sites for flippase based marker excision of the Tetracycline resistance (TetR) cassette with the regulatory and reporter modules contained in the shaded region denoted in panel E. (**D and E**) Comparative schematics of regulatory and reporter modules of the cumate circuits, illustrating P_pc_/CymR and optimized P_CymRC_/CymR_GV_ configurations. Within CymR_GV_ the amino acid substitutions contained in this variant are denoted in pink and blue with additional elements including the multiple cloning sites (MCS), Cumate operators (CuO), Hammerhead Ribozyme (HHRz) components, as well as the His terminator also denoted.

**Fig 2:**
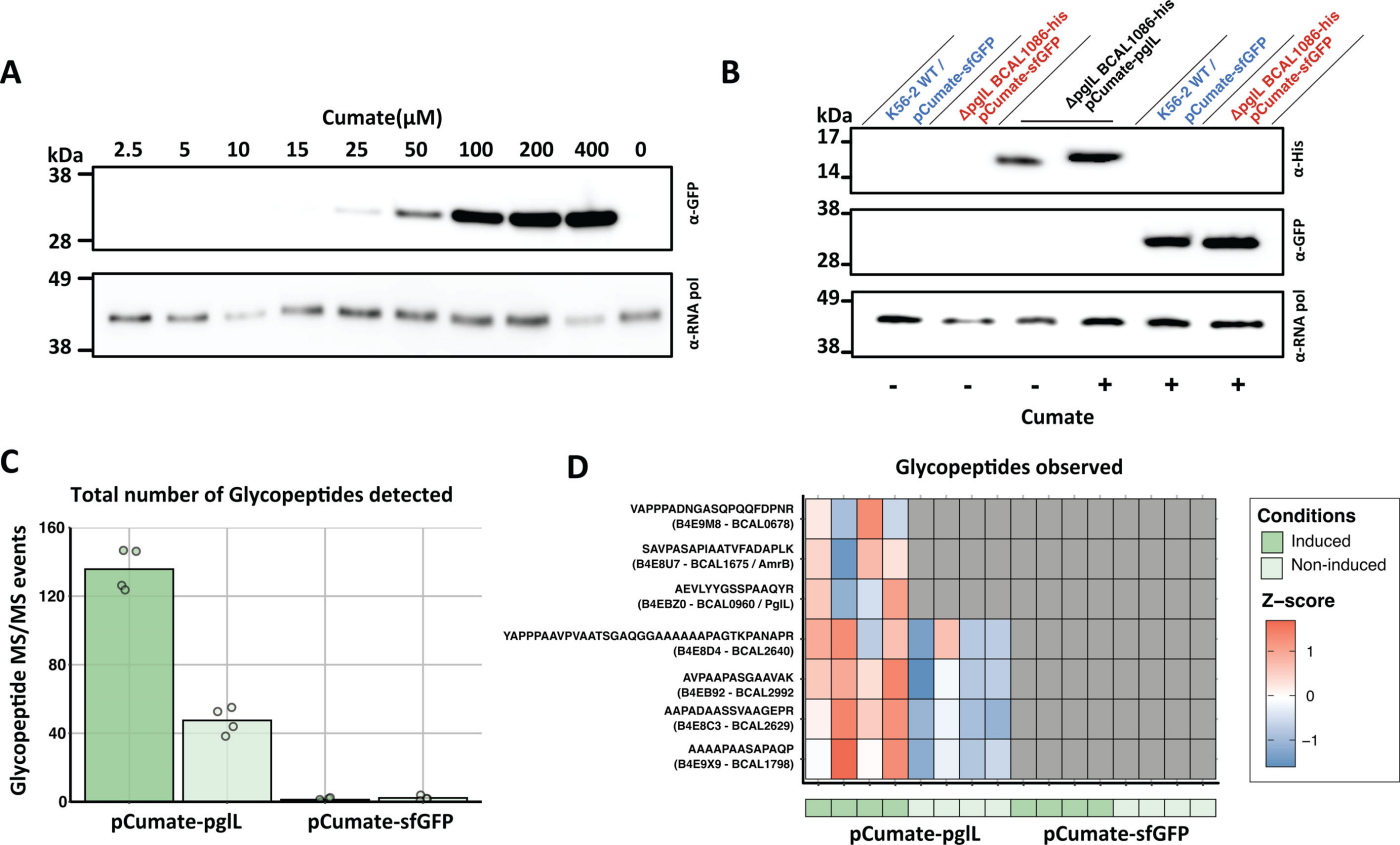
The cumate circuit P_Cym_/CymR displays high basal expression in *B. cenocepacia*. (**A**) Western blot analysis demonstrates tuneable expression of sfGFP from pCumate-sfGFP in response to cumate. (**B**) Immunoblot analysis of BCAL1086-his and sfGFP expression within the Δ*pglL* BCAL1086-his reporter strain revealing glycosylation in the absence of induction in a pCumate-PgIL_Bc_-his dependent manner. (**C**) Quantification of glycopeptides identified from whole cell proteomic analysis of *B. cenocepacia* Δ*pglL* BCAL1086-his containing pCumate-PglL*_Bc_*-his or pCumate-sfGFP with and without induction with 100 μM cumate (*n* = 4). (**D**) Heatmap of selected glycopeptides observed altered in *B. cenocepacia* Δ*pglL* BCAL1086-his reveals glycosylation in the absence of induction which is enhanced upon cumate induction (*n* = 4 per group).

To compare the available cumate regulatory systems, we first integrated the canonical circuit P_Cym_/CymR into the broad-host pBBR1-based vector pMLBAD (35), generating the plasmid pCumate-sfGFP (Fig. 1A and D). In *B. cenocepacia* K56-2, we assessed sfGFP expression from pCumate-sfGFP, demonstrating dose-dependent sfGFP production by western blotting (Fig. 2A). While fluorescent reporter proteins provide a convenient method to assess protein induction, these systems can mask basal expression, which may occur below the dynamic range detectible by western blotting or direct fluorescent assessment. Based on this, we reasoned that the use of an enzymatic reporter may provide a more sensitive read-out for basal activity. We, therefore, selected the *B. cenocepacia* protein glycosylation oligosaccharyltransferase PglL (BCAL0960) (44), as a model, which have previously shown to produce robust glycosylation at low levels of protein expression (43). Placing PglL under cumate inducible control, we generated pCumate-PglL*_Bc_*-his and assessed the restoration of glycosylation in the *B. cenocepacia* strain Δ*pglL* BCAL1086-his (45). In this background, the periplasmic glycoprotein BCAL1086 has been chromosomally his-tagged, with prior studies demonstrating the loss of this protein in the absence of glycosylation, providing a sensitive readout for PglL activity (43, 45). Introduction of pCumate-PglL*_Bc_*-his into Δ*pglL* BCAL1086-his resulted in the appearance of BCAL1086-his in the absence of induction, with the addition of cumate resulting in a decrease in gel mobility consistent with inducer-dependent glycosylation (Fig. 2B). Glycoproteomic analysis confirmed the introduction of pCumate-PglL*_Bc_*-his in Δ*pglL* BCAL1086-his partially restored glycosylation in the absence of induction (Fig. 2C and Table S5) with induction also observed to enhance glycosylation in a pCumate-PglL_Bc_-his-dependent manner, as determined by the quantitative glycopeptides analysis (Fig. 2D and Table S6). Taken together, we conclude that while P_Cym_/CymR is functional in *B. cenocepacia* it displays high basal expression, as demonstrated by the detection of protein glycosylation in the absence of induction in a glycosylation-null complemented background.

### Combining CymR circuit features reduces basal expression

**Fig 3:**
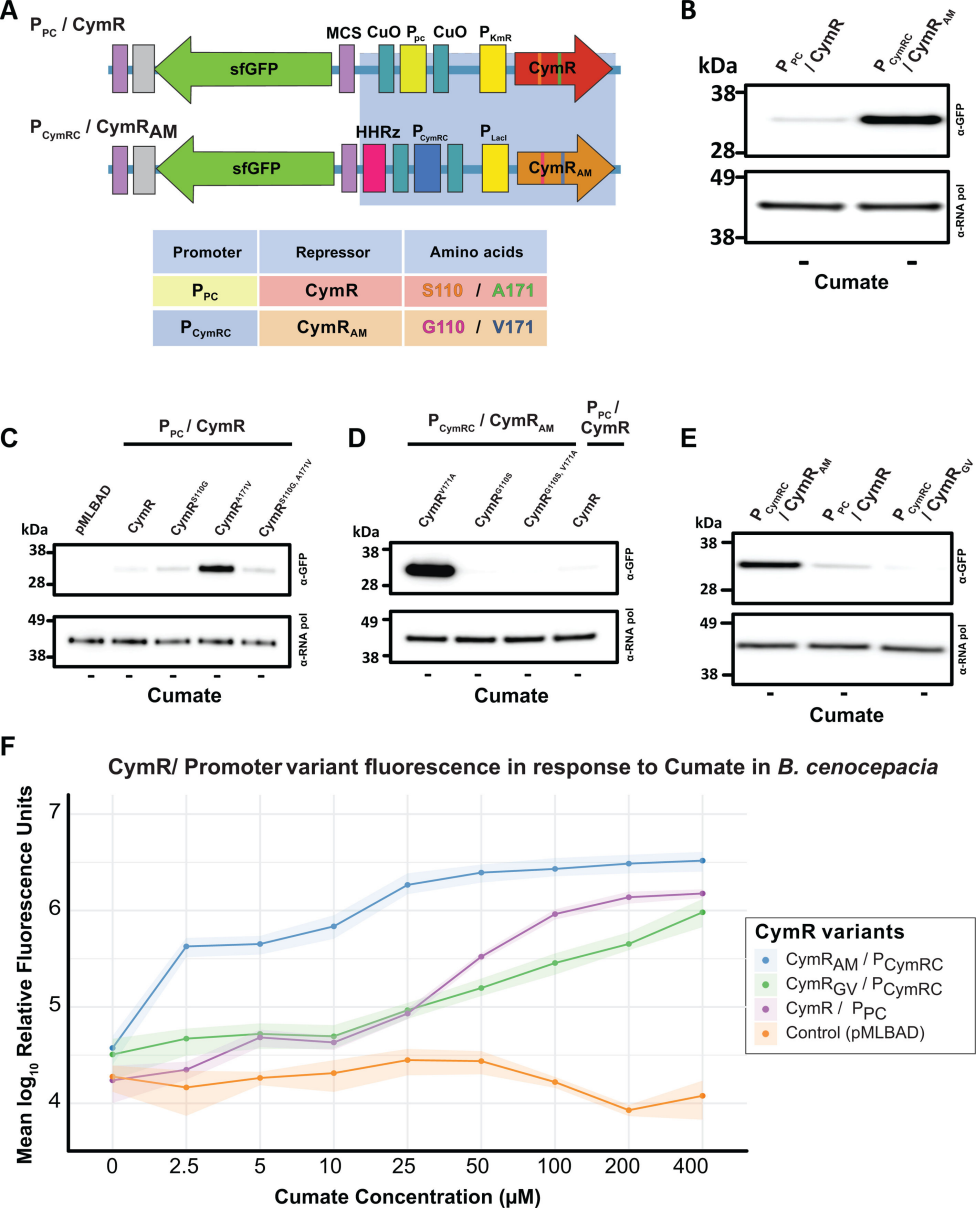
Assessment of basal expression across cumate circuits and the generation of the p_CymRC_/CymR_GV_ cumate circuit. (**A**) Schematic comparison of cumate regulatory circuits P_pc_/CymR (top) and P_CymRC_/CymR_AM_ (bottom) with the amino acid differences between these variants highlighted. (**B**) Comparison of basal sfGFP expression between P_CymRC_/CymR_Am_ and the P_PC_/CymR variants. (**C**) Western blot analysis of sfGFP expression using the wild-type repressor P_PC_/CymR and its variants, revealing that the introduction of S110G (orange) or A171V (green) substitutions increase basal expression of sfGFP. (**D**) Immunoblot analysis of CymRAm derivatives, revealing that the reversion of G110S (pink) or V171A (blue) amino acid substitutions reduces basal expression of sfGFP. (**E**) sfGFP basal expression observed across the three cumate circuits, P_CymRc_/CymR_Am_, P_PC_/CymR, and p_CymRC_/CymR_GV_, reveals that P_CymRC_/CymR_GV_ demonstrates the lowest levels of basal expression. (**F**) Line graph of sfGFP fluorescence at 24-h post-induction with different cumate concentrations. The standard deviation is represented by the shaded areas with individual data points corresponding to the mean of three independent biological replicates.

As previous studies have demonstrated the functionality of P_CymRC_/CymR_AM_ in *Burkholderia thailandensis* (27), we assessed the performance of this circuit in *B. cenocepacia*. Within this circuit, CymR_AM_ corresponds to an evolved CymR variant obtained through directed evolution by Adam Meyer (AM) and colleagues, which has previously been suggested to possess an improved dynamic range and specificity. To allow a fair comparison of these circuits, we exchanged P_Cym_/CymR in pCumate-sfGFP with P_CymRC_/CymR_AM_ from pKCyR5 (27), generating pCumate_AM_-sfGFP (Fig. 3A). Surprisingly, we observed an increase in sfGFP expression when placed under P_CymRC_/CymR_AM_ control, compared to P_Cym_/CymR, in the absence of induction in *B. cenocepacia* (Fig. 3B). We initially reasoned that the expression changes due to codon usage in CymR_AM_, which was codon optimized for *E. coli* (3), may account for this increased expression. To assess this, we reintroduced the two amino acid substitutions from CymR_AM_, Ser110Gly (S110G), and Ala171Val (A171V) into the canonical CymR sequence in P_Cym_/CymR generating CymR^S110G^, CymR^A171V^, and CymR^S110G,A171V^. These two mutations, either individually or together, resulted in increased basal sfGFP expression with the alteration of position A171V leading to the largest increase in expression, which was reduced by co-introduction of S110G (Fig. 3C). These trends were also observed in *E. coli* (Fig. S1A and B) suggesting the prior reported alterations in CymR_AM_ are associated with unexpected effects in the absence of induction. To confirm this hypothesis, we reverted the mutations within CymR_AM_ to the canonical amino acids, generating CymR_AM_^G110S^, CymR_AM_^V171A^, and CymR_AM_^G110S,V171A^. As with CymR, the alteration of position 171 led to a marked increase in basal expression which was suppressed by the alteration of position 110 (Fig. 3D). Assessment of CymR_AM_, CymR, and CymR_AM_^G110S,V171A^ revealed CymR_AM_^G110S,V171A^ resulted in reduced basal expression (Fig. 3E), with this reduction also observed in *E. coli* (Fig. S1C). These results revealed that CymR_AM_^G110S,V171A^, herein referred to as CymR_GV_ (Fig. 1B and E), in conjunction with the promoter P_CymRC_ reduces basal expression, as assessed by sfGFP production, compared to P_Cym_/CymR and P_CymRC_/CymR_AM_ circuits in *B. cenocepacia*. To investigate sfGFP expression in an orthogonal manner, we assessed the fluorescence of sfGFP under the control of P_Cym_/CymR, P_CymRC_/CymR_AM_, and P_CymRC_/CymR_GV_ circuits. We observed that cumate circuits possessing identical CymR amino acid sequences, P_Cym_/CymR and P_CymRC_/CymR_GV_, resulted in similar kinetics in *B. cenocepacia,* while P_CymRC_/CymR_AM_ showed heightened responsiveness to cumate even at low levels of induction (Fig. 3F) with these trends also observed in *E. coli* (Fig. S2). Together, these results suggest that the use of P_CymRC_/CymR_AM_ leads to increased basal expression and narrower responsiveness to cumate compared to P_Cym_/CymR. These effects were reversed by the restoration of amino acid substitutions in the CymR_AM_ sequence to its canonical form leading to reduced basal expression and widening of the cumate responsiveness.

### P_CymRC_/CymR_GV_ improves tuneable control of protein expression in *B. cenocepacia*

**Fig 4:**
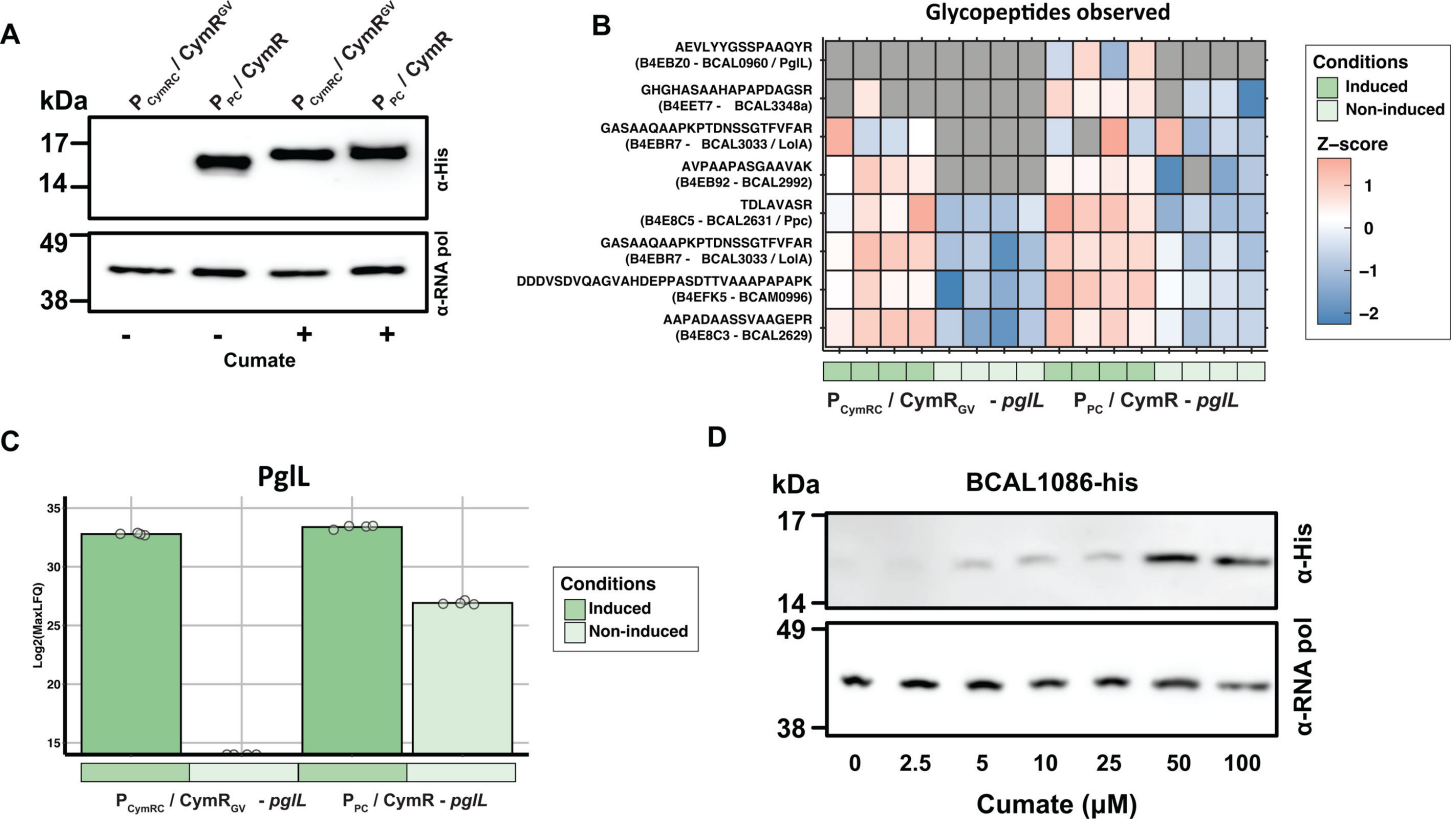
P_CymRC_/CymR_GV_ reduces basal expression, allowing inducible control of protein glycosylation. (**A**) Immunoblot of BCAL1086-his supports improved control of protein glycosylation, with BCAL1086-his only detected upon induction of PglL-his under p_CymRC_/CymR_GV_ compared to P_pc_/CymR. (**B**) Heatmap of selected glycopeptides observed within *B. cenocepacia* Δ*pgl*L BCAL1086-his, demonstrating reduced but detectable glycosylation from PglL under p_CymRC_/CymR_GV_ control compared to P_pc_/CymR (*n* = 4 per group). (**C**) Quantification of PglL protein levels from whole-cell proteomic analysis reveals PglL is undetectable when under p_CymRC_/CymR_GV_ control in the absence of induction. (**D**) Immunoblot of BCAL1086-his demonstrating the dose-dependent induction of BCAL1086-his expression with increasing cumate concentrations.

The reduced basal expression of sfGFP indicated tighter regulatory control from P_CymRC_/CymR_GV_ compared to other assessed cumate circuits. To directly assess this in a biologically relevant context, we placed PglL under P_CymRC_/CymR_GV_ control, generating pCumate_GV_-PglL_Bc_-his, and again introduced this into *B. cenocepacia* Δ*pgl*L BCAL1086-his. Consistent with reducing basal expression of PglL, western analysis revealed the absence of detectible BCAL1086-his without induction in strains containing pCumate_GV_-PglL_Bc_-his (Fig. 4A). Upon cumate induction, we observed the appearance of BCAL1086-his at a similar mobility to BCAL1086-his observed with pCumate-PglL_Bc_-his, indicating the initiation of glycosylation (Fig. 4A). Quantitative glycoproteomics/proteomics confirmed the induction of glycosylation in response to cumate in *B. cenocepacia* Δ*pglL* BCAL1086-his containing pCumate_GV_-PglL_Bc_-his and pCumate-PglL_Bc_-his (Fig. 4B). Glycoproteomic analysis also revealed the presence of low-level glycosylation even in the absence of cumate induction (Fig. 4B**,** Fig. S3 and Table S7). Proteomic analysis reveals the lack of detectable PglL in strains containing pCumate_GV_-PglL_Bc_-his, compared to pCumate-PglL without induction (Fig. 4C and Table S8). Combined, the absence of detectible BCAL1086-his and PglL in *B. cenocepacia* Δ*pglL* BCAL1086-his containing pCumate_GV_-PglL supports reduced basal expression of PglL from P_CymRC_/CymR_GV_ and that P_CymRC_/CymR_GV_ achieves titratable modulation of glycosylation, as demonstrated by the inducible detection of BCAL1086-his in response to increasing cumate levels (Fig. 4D). These results support the notion that P_CymRC_/CymR_GV_ allows tuneable protein expression in *Burkholderia*.

Previous work in our lab has shown that commonly used *Burkholderia* spp. expression systems can drive large proteomic alterations in *B. cenocepacia* (43). To understand the effects of cumate-based induction on the *B. cenocepacia* proteome, we undertook Data-Independent Acquisition (DIA) based proteomics. To allow assessment of cumate-based induction on the *B. cenocepacia* proteome without potentially confounding effects of sfGFP within pCumate_GV_-sfGFP, we exchanged sfGFP with a multi-cloning site, generating pCumate_GV_-MCS. To benchmark the proteome changes observed in response to pCumate_GV_-MCS, we selected the widely employed rhamnose-inducible *B. cenocepacia* expression plasmid pSCrhaB2 (36) as a comparator. As an additional control, the vector pBBR1-EV was constructed possessing an identical backbone to pCumate_GV_-MCS and pSCrhaB2 yet lacking both the cumate- and rhamnose-inducible circuits. Using these vectors, the impact of induction with 100 μM cumate on *B. cenocepacia* containing pCumate_GV_-MCS and pBBR1-EV, as well as the impact of 0.5% L-rhamnose on *B. cenocepacia* containing pSCrhaB2 and pBBR1-EV, was assessed leading to the identification of 4,294 proteins (Table S9). To allow assessment of the impact of plasmids in the absence of induction, non-induced controls were included for each plasmid group, and all biological replicates grown under antibiotic selection.

**Fig 5:**
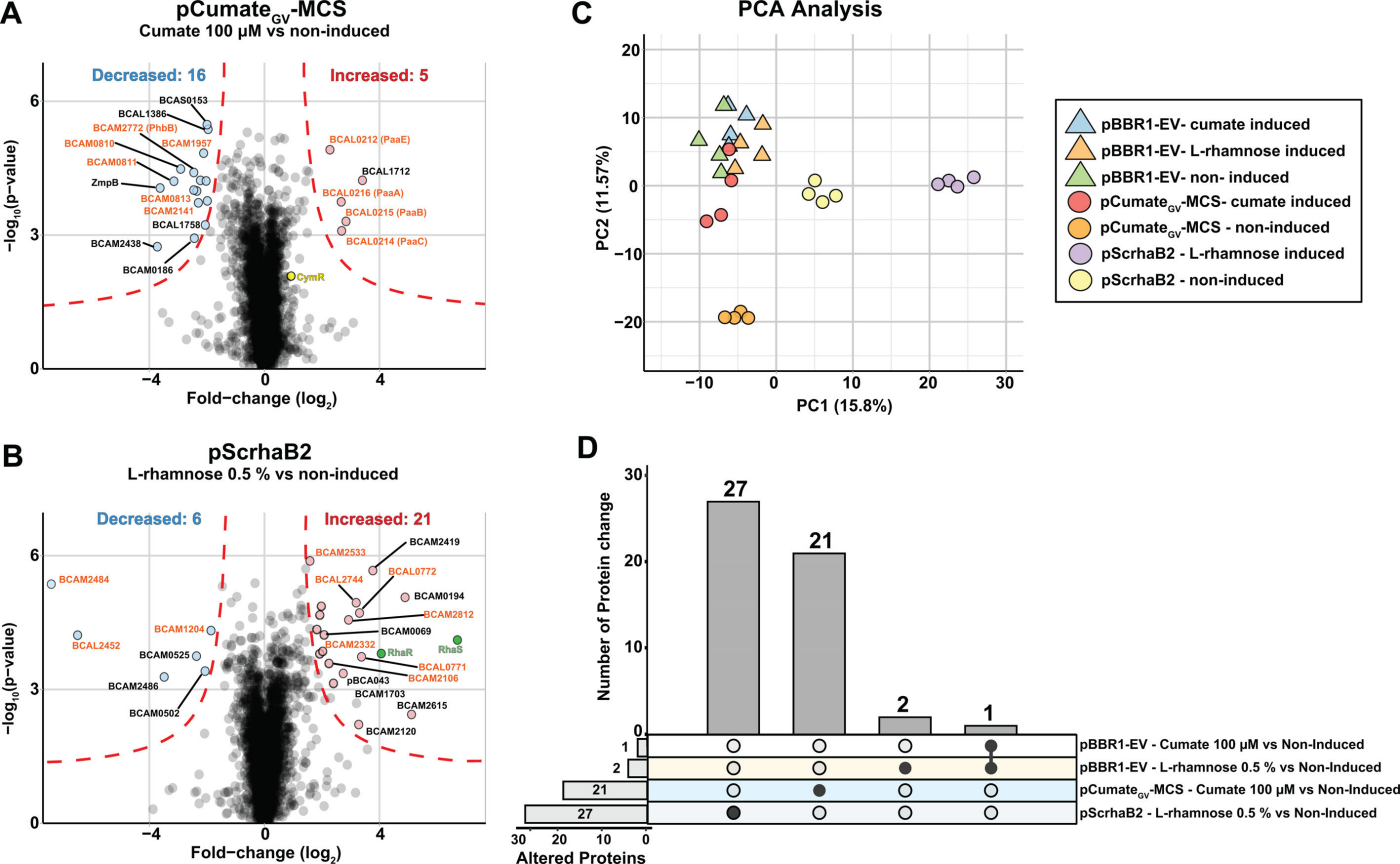
Proteomic analysis of cumate and rhamnose induction within *B. cenocepacia*. (**A and B**) Volcano plots illustrating the differential protein abundance profiles between induced and non-induced conditions for cumate using pCumate_GV_-MCS (**A**) and rhamnose control using pSCrhaB2 (**B**). Significance thresholds are denoted by the red S0 lines with altered proteins labeled. Proteins of interest are denoted in brown, with the regulatory proteins RhaS/R denoted in green and CymR in orange. (**C**) Principal component analysis (PCA) of all samples, showing clear separation of induced pCumate_GV_-MCS and pSCrhaB2 groups from their uninduced counterparts and pBBR1-EV controls. (**D**) Upset plot showing the number of proteins exhibiting significant alterations under each condition, with overlap analysis highlighting shared and unique proteomic changes associated with cumate and rhamnose induction.

A total of 21 protein alterations were observed in response to cumate induction in *B. cenocepacia* harboring pCumate_GV_-MCS (Fig. 5A), with reductions observed in the polyhydroxybutyrate synthase PhbB (BCAM2772), the zinc metalloprotease ZmpB (BCAM2307), the aromatic compound catabolism-associated proteins BCAM0810, BCAM0811, and BCAM0813, and the ABC transporters BCAM2141 and BCAM1957. Cumate induction also led to increased abundance of several phenylacetic acid (PAA) degradation–cluster proteins, including BCAL0212 (PaaE), BCAL0214 (PaaC), BCAL0215 (PaaB), and BCAL0216 (PaaA), suggesting alterations in aromatic degradation pathways in response to the presence of pCumate_GV_-MCS and cumate induction. In contrast, rhamnose induction in strains harboring pSCrhaB2 resulted in a total of 27 protein alterations (Fig. 5B), including changes in several transcriptional regulators within increases in the LysR-family regulators BCAM2332 and BCAM2812, the GntR-family regulator BCAL2744 and the AraC-family regulator BCAL0772, and a decrease in the LysR-family regulator BCAL2452. These regulatory changes were accompanied by shifts in metabolic and stress-related enzymes, with increased abundance of the two-component regulatory kinase BCAM2533, glutathione peroxidase BCAM2106 and the predicted non-heme chloroperoxidase BCAL0771, and reductions in BCAM1204 (encoding the alanine racemase DadX) as well as BCAM2484 (MetE). These results suggest that pCumate_GV_-MCS produces comparable levels of proteomic perturbation to the widely used pSCrhaB2 expression system.

To assess trends within the proteome across samples, principal-component analysis (PCA) was undertaken, revealing clear clustering of biological groups, yet separation of pSCrhaB2 and the non-induced pCumate_GV_-MCS groups from all other samples (Fig. 5C). We observed that induction with either cumate or rhamnose did not cause pBBR1-EV-associated samples to separate and, consistent with this, only modest protein alterations were induced in strains containing pBBR1-EV in response to induction (Fig. S4). These modest alterations suggest that separation within the PCA is driven by the combination of inducer and the presence of plasmids bearing compatible inducible circuits. In line with this, analysis of the proteins altered in response to pCumate_GV_-MCS induction, relative to pBBR1-EV indicates that CymR_GV_ expression alone modulates *B. cenocepacia* proteome, with these effects reversed upon cumate addition (Fig. S5A). In contrast, induction of rhamnose-inducible circuits drives the observed proteomic effects, leading to proteomic states that differ from those seen with pBBR1-EV in response to rhamnose induction (Fig. S5B). These trends indicate that cumate and rhamnose-inducible systems exert distinct, orthogonal effects on the *B. cenocepacia* proteome with minimal overlap (Fig. 5D). Taken together, these findings indicate that the presence of CymR_GV_ can alter the *B. cenocepacia* K56-2 proteome, yet these effects are suppressed upon induction with cumate. Importantly, suppression of inducer circuit-associated proteomic effects is not observed with the widely used rhamnose-inducible plasmid pSCrhaB2, highlighting differences in the mechanisms of protein induction between these systems.

### Chromosomal P_CymRC_/CymR_GV_ allows inducible control within an *in vivo* context revealing the requirement of protein O-linked glycosylation for optimal intracellular replication

**Fig 6:**
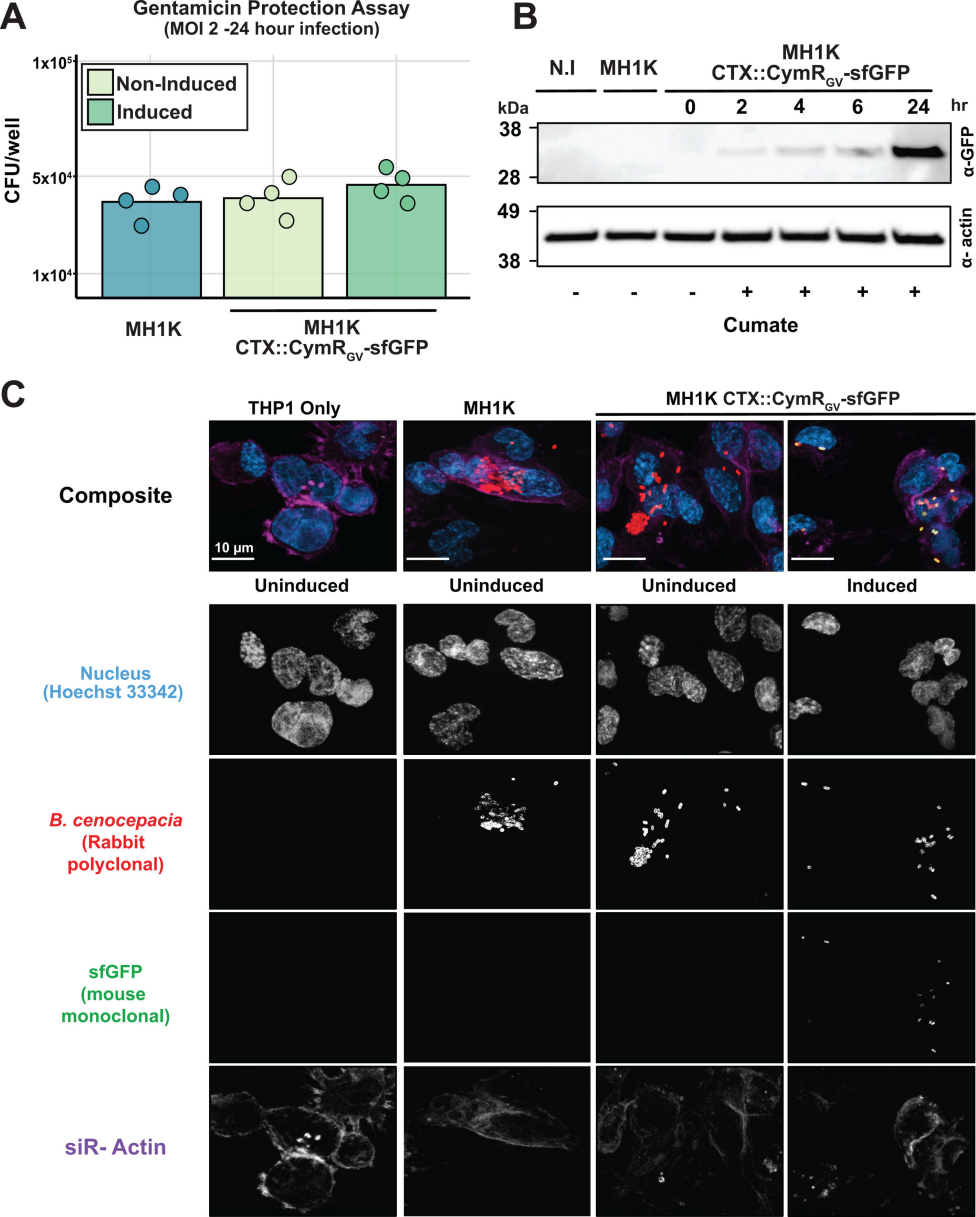
Cumate induction allows inducible control of protein expression from *B. cenocepacia* within THP-1 cells. (**A**) Gentamicin protection assays quantifying intracellular bacterial burden at 24 h post-infection (MOI 2). CFU per well recovered from THP-1 cells infected with wild-type MH1K or MH1K CTX::CymR_GV_-sfGFP under non-induced and cumate-induced conditions demonstrate comparable intracellular survival. (**B**) Immunoblot of sfGFP following cumate induction (0, 2, 4, 6, and 24 h) in intracellular MH1K CTX::CymR_GV_sfGFP. sfGFP is detectable by 2 h and increases over time. α-actin serves as a loading control. N.I., non-infected THP-1 cells. (**C**) Immunofluorescence microscopy of internalized MH1K and MH1K containing CymR_GV_-sfGFP strains in THP-1 cells under induced and uninduced conditions reveal co-localisation of sfGFP and *B. cenocepacia* in an induction dependent manner. DNA has been stained with Hoechst 33,342, *B. cenocepacia* with Alexa Fluor 568, sfGFP visualized through intrinsic fluorescence, and actin filaments labeled with SiR-actin 647. Scale bars, 10 μm.

**Fig 7:**
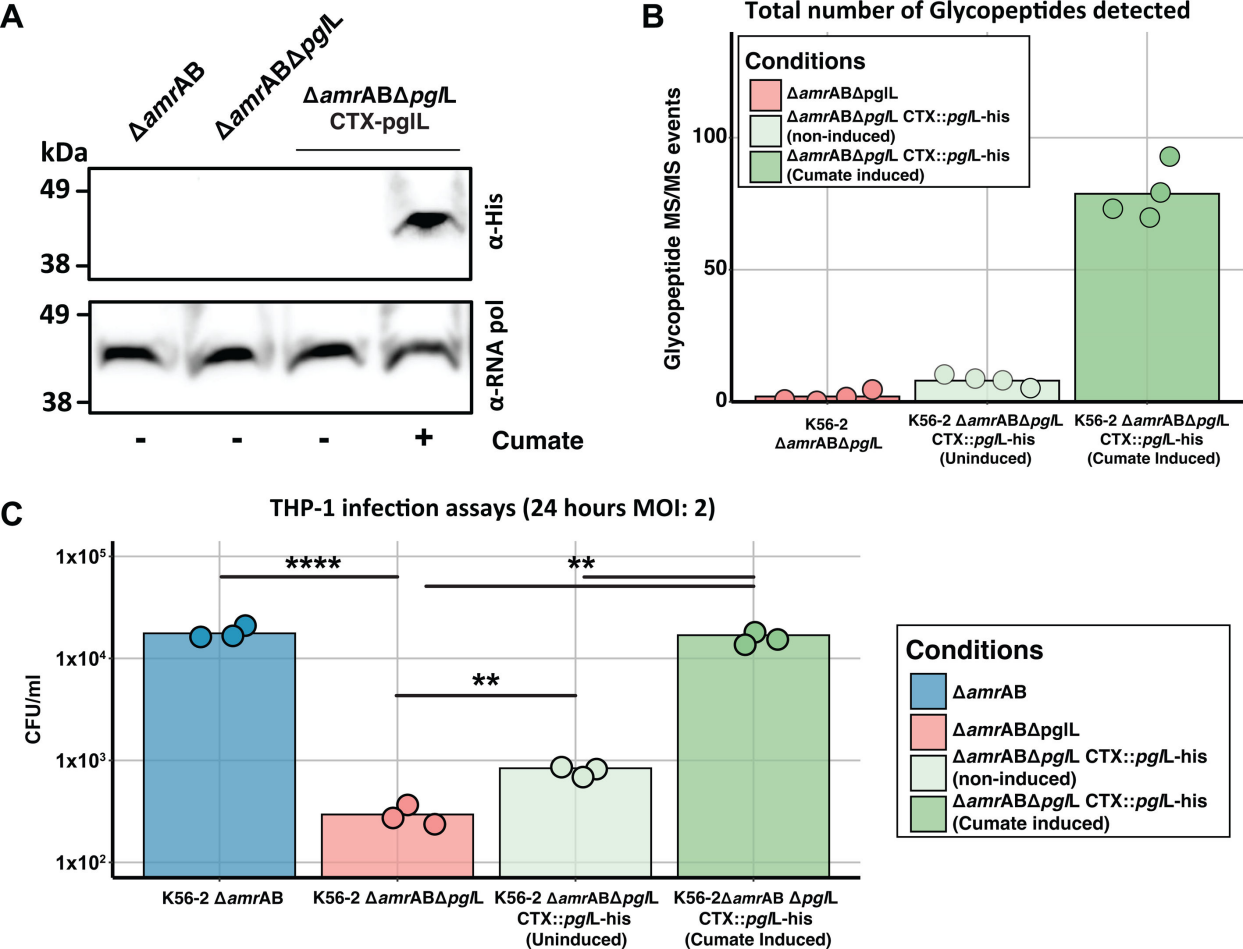
Protein glycosylation contributes to intracellular replication of *B. cenocepacia* within THP-1 cells. (**A**) Immunoblot of His-tagged PglL in *B. cenocepacia* K56-2 Δ*amrAB*Δ*pglL* reveals induction dependent detection of PglL-his. (**B**) Quantitation of glycopeptides identified by glycoproteomic analysis of *B. cenocepacia* K56-2 Δ*amr*ABΔ*pgl*L complemented with CymR_GV_-PglL_Bc_-his. Glycopeptides are undetectable in the Δ*amr*ABΔpglL mutant with cumate induction allowing the restoration glycosylation. (**C**) Intracellular survival of *B. cenocepacia* K56-2 Δ*amrAB* strains in THP-1 macrophages at 24 h post-infection (MOI = 2). Δ*amr*ABΔ*pgl*L exhibits reduced survival relative to Δ*amr*AB, while cumate induced pglL-his complementation restored intracellular burden to near wild-type levels upon induction. Each point corresponds to the mean of four technical replicates for a given biological replicate. ****P < 0.0001, **P < 0.005.

While the plasmid-based P_CymRC_/CymR_GV_ expression system enables tightly regulated protein induction in *B. cenocepacia*, the requirement for continuous plasmid selection is not achievable in all contexts such as during infection studies. To overcome these limitations, chromosomal integration provides an alternative strategy to allow tuneable expression under *in vivo* conditions without the need for continuous selection. To assess the viability of using P_CymRC_/CymR_GV_ for *in vivo* chromosomal based protein expression, the P_CymRC_/CymR_GV_ circuit was incorporated into a Mini-CTX1 integration vector to facilitate single-copy insertion at the *attB* locus in *B. cenocepacia* (40, 46), generating pCTX-CymR_GV_-sfGFP and pCTX-CymR_GV_-MCS (Fig. 1C). Using the Gentamicin-sensitive *B. cenocepacia K56-2* strain MH1K (47), pCTX-CymR_GV_-sfGFP was integrated into the chromosome to generate MH1K CTX-sfGFP with the effects on replication assessed against MH1K. Intracellular replication was assessed within THP-1 cells using OD_600_ normalized bacterial inoculum (Fig. S6) at a multiplicity of infection (MOI) of 2, with intracellular bacterial burden assessed 24-h post-infection. A 24-h time point was selected, in line with prior work demonstrating robust *B. cenocepacia* replication within macrophage models under these conditions, enabling defects in intracellular survival to be readily identified (47). No significant difference in bacterial colony forming units (CFUs) was observed between MH1K CTX-sfGFP with or without cumate induction compared to MH1K alone (Fig. 6A). Western blot analysis of infected THP-1 cells shows controlled induction of sfGFP, which is detectable as early as 2 h after intracellular induction (Fig. 6B). Utilizing confocal immunofluorescence microscopy, we assessed the internalization of *B. cenocepacia* within THP-1 cells as well as the colocalization of sfGFP within *B. cenocepacia* in response to cumate induction post internalization (Fig. 6C). These observations indicate that chromosomally integrated CTX P_CymRC_/CymR_GV_ allows inducible expression of proteins in *B. cenocepacia* K56-2 during intracellular replication. Finally, to further demonstrate the capacity of pCTX-CymR_GV_ to facilitate intracellular studies of *B. cenocepacia*, we placed PglL*_Bc_*-his in pCTX-CymR_GV_ and integrated this into a Gentamicin-sensitive glycosylation-null strain, *B. cenocepacia* K56-2 Δ*amr*ABΔ*pglL*. Inducible expression of PglL*_Bc_*-his from chromosomally integrated CTX-CymR_GV_-PglL*_Bc_*-his was confirmed by western blotting (Fig. 7A) and the successful restoration of glycosylation by glycoproteomic analysis (Fig. 7B, Tables S10 and Table S11). Utilizing Δ*amr*ABΔ*pglL* CTX-CymR_GV_-PglL*_Bc_*-his, we assessed the intracellular burden of this strain within THP-1 cells 24-h post infection compared to Δ*amr*AB and Δ*amr*ABΔ*pglL* using OD_600_ normalized infections (Fig. S7). CFU counts reveal a reduction in the bacterial load of Δ*pgl*L strains compared to Δ*amr*AB, with induction of PglL_Bc_-his in Δ*amr*ABΔ*pglL* CTX-CymR_GV_-PglL_Bc_-his restoring bacterial CFUs to parental levels (Fig. 7C). Combined, these findings support that PglL*_Bc_*-his contributes to the ability of *B. cenocepacia* to replicate in THP-1 cells and demonstrates the ability of our CTX cumate-inducible system to facilitate inducible *in vivo* studies in *B. cenocepacia*.

## DISCUSSION

Inducible expression vectors enable controlled gene expression and are indispensable tools for molecular microbiology (2, 48). While carbohydrate-inducible systems are widely employed in *B. cenocepacia*, these systems can be associated with several shortcomings, including modulating central metabolism and inducing stress responses (36, 42, 49). To address these limitations, we have assessed and refined cumate-inducible systems for *B. cenocepacia* (Fig. 1). While previous work demonstrated the functionality of the cumate-inducible system P_CymRC_/CymR_AM_ in *Burkholderia* (27), we find that available cumate circuits (P_Cym_/CymR & P_CymRC_/CymR_AM_) both possess high levels of basal expression, limiting their utility and motivating us to improve the available circuits which are utilized for cumate induction. Critically, we find that while the functionality of cumate circuits can be assessed using reporter proteins by western or fluorescent assays (Fig. 2 and Fig. 3), these approaches are largely insufficient to gage basal expression. As has been exploited to allow highly sensitive detection of protein translocation (50, 51), we observed that the use of an enzymatic process, in this case protein glycosylation, provides a more sensitive and meaningful way to track basal expression in *B. cenocepacia,* allowing us to refine a new cumate circuit (P_CymRC_/CymR_GV_) utilizing western-based analysis with the glycosylation-sensitive protein BCAL1086-his (43, 45) and glycoproteomics.

Our observations of high basal expression utilizing the P_Cym_/CymR circuit are in line with prior reports noting challenges with this inducible system when manipulating deleterious enzymatic processes (52, 53). Pöschel et al. noted that in *Methylorubrum extorquens*, the use of P_Cym_/CymR for the expression of a recombinant mevalonate pathway resulted in growth abnormalities, which were only suppressed by a spontaneous mutation in the promoter P_Cym_, reducing basal expression (53). Our work supports that while P_Cym_/CymR is functional in *B. cenocepacia*, it does not provide sufficient control to limit basal expression (Fig. 2). While reducing basal expression motivated us to explore the use of P_CymRC_/CymR_AM_, which was developed to possess improved characteristics (3), we find that this circuit, both in *B. cenocepacia* (Fig. 3) and *E. coli* (Fig. S1 and S2), displays shallower cumate responsiveness and higher levels of basal expression compared to the canonical P_Cym_/CymR circuit. These characteristics can be reverted by restoring CymR_AM_ to the progenitor CymR sequence (Fig. 3 and Fig. 4), highlighting an effective means to improve basal control in vectors that have utilized P_CymRC_/CymR_AM_. Utilizing the P_CymRC_/CymR_GV_ circuit, we find that tight expression control can be achieved from both plasmid (Fig. 4) and chromosomally integrated (Fig. 6 and Fig. 7) vectors in *B. cenocepacia*. While our work has predominantly focused on *B. cenocepacia*, these systems are equally amenable to other members of the *Burkholderia* genus, including *Burkholderia thailandensis*, where we have confirmed the functionality of the P_CymRC_/CymR_GV_ circuit (Fig. S8). Importantly, it should be noted that while many *Burkholderia* species lack pathways for the catabolic metabolism of cumate, several species do possess cym pathways which have been shown to be functional, such as *Burkholderia xenovorans* (54, 55). Thus, cumate induction and responsiveness should be assessed on a case-by-case basis when applying these systems to diverse *Burkholderia* members as the assumption that cumate is non-metabolizable may not hold true for all species.

A key motivation for refining cumate-inducible systems for use in *Burkholderia* was driven by our observation of the proteomic consequences of rhamnose induction (43). Consistent with our previous observations, rhamnose induction in *B. cenocepacia* containing pSCrhaB2 drives distinct remodeling of the proteome compared with cumate (Fig. 5A through D). While this is expected, our proteomic analysis of pBBR1-EV in response to inducers supports it is not the inducers themselves that drive these effects, but rather the combination of the regulatory circuits and inducers. We hypothesize that differences in how protein induction is controlled between the cumate and rhamnose circuits may account for the observed proteomic trends. Critically, the addition of cumate suppresses DNA binding of CymR_GV_ (20) and, in line with this, we observe restoration of the proteome to a state similar to that seen in the absence of this regulator, akin to pBBR1-EV (Fig. 5C and Fig. S5A). In contrast, rhamnose-based induction appears to drive proteomic alterations, which is consistent with rhamnose induction initiating RhaS DNA binding to drive protein expression (56, 57) (Fig. 5C and Fig. S5B). Our proteomic results indicate that, although regulatory circuits such as the cumate- and rhamnose-inducible systems are typically considered inert, they have noticeable impacts on the *B. cenocepacia* proteome. This supports the idea that the underlying mechanisms by which gene expression is controlled—either by blocking expression in the absence of induction, as in the case of CymR, or by driving DNA binding in response to an inducer, as in the case of RhaS—should be considered when selecting systems for tuneable protein expression. Interestingly, many observed protein changes upon cumate induction appear to be associated with a reduction in protein levels, including reductions in known virulence factors such as zmpB (BCAM2307). While this suggests that the addition of cumate may suppress potential virulence pathways in *B. cenocepacia*, this did not lead to alterations in intracellular survival within strains containing chromosomally integrated P_CymRC_/CymR_GV_ vectors (Fig. 6A and Fig. 7C). Critically, as cumate is cell-permeable, we demonstrate that this provides the unique ability to trigger protein induction during *in vivo* studies (Fig. 6C). While approaches for intracellular expression have been developed for other bacterial pathogens, such as *Salmonella*, where promoters associated with the *Salmonella* pathogenicity island 2 have been used for temporal protein control (58, 59), these systems are not generalizable across bacterial species. To our knowledge, this is the first example of using cumate-induced systems for titratable protein expression control within an intracellular bacterial infection model. Overall, while cumate induction has been used for eukaryotic expression systems (26), its ability to allow expression induction in an infection context expands the field’s capacity to study intracellular pathogens such as *B. cenocepacia*.

Finally, we demonstrate that the loss of glycosylation reduces the bacterial burden in a THP-1 infection model, and utilizing our refined cumate-inducible system, this effect can be reversed upon complementation (Fig. 7C). This finding is consistent with a growing body of evidence that *Burkholderia* O-glycosylation contributes to fitness and pathogenicity (60, 61). Interestingly, while protein glycosylation has been shown by several teams to contribute to *B. cenocepacia* survival in *Galleria mellonella* (62, 63), the observation of its role in mammalian intracellular replication has not been previously demonstrated. Recently, Paszti et al. demonstrated using Tn-Seq that several genes associated with protein glycosylation glycan biosynthesis (BCAL3114 & BCAL3115) were essential in both *G. mellonella* and *ex vivo* pig lung models; however, they only observed the requirement for PglL_Bc_ (BCAL0960) in *G. mellonella* (62). Our observation that the lack of PglL_Bc_ reduces but does not abolish the recovery of *B. cenocepacia* from THP-1 suggests that O-linked glycosylation contributes to survival but may not be essential for pathogenesis *per se*. Utilizing this inducible system, future work will seek to understand how the absence of glycosylation shapes the course of intracellular infections and if the lack of glycosylation simply reduces bacterial replication or impacts specific processes during intracellular survival, leading to the observed reduction in bacterial load.

Combined, this work establishes a set of refined cumate-inducible vectors for *B. cenocepacia*, allowing tight inducible control as well as the capacity to induce protein expression within *in vivo* models. While the focus of this work sought to expand the availability of inducible systems for *Burkholderia* studies, these cumate vectors are likely applicable to other non-model bacteria where gene control can be challenging. In summary, by refining and developing novel cumate-inducible vectors, this work expands the genetic toolkit for studying the *Burkholderia* genus.

## MATERIALS AND METHODS

### Bacterial strains, plasmids, and growth conditions

The bacterial strains utilized in this study are detailed in Table S1. The plasmids used in this study are listed in Table S2. Strains were cultured in Lysogeny Broth (LB- BD, USA) or on LB agar (1.5%–2%, wt/vol), prepared according to the manufacturer’s instructions and containing 0.5% NaCl. Liquid cultures were incubated overnight at 37°C with 180 rpm shaking, whereas agar plates were incubated at 37°C overnight for *E. coli* and for 24–72 h for *B. cenocepacia*. Antibiotics were incorporated into cultures to select plasmids/transconjugants at a final concentration of 50 μg/mL for *E. coli*, 100 μg/mL for *B. cenocepacia for* Trimethoprim; 20 μg/mL for *E. co*li, 150 μg/mL for *B. cenocepacia for* Tetracycline as well as 50 μg/mL Kanamycin and 10 μg/mL Gentamicin for *E. coli*. To selectively inhibit helper and donor *E. coli* strains after triparental mating, Ampicillin was added to agar plates at a concentration of 100 μg/mL and Polymyxin B at 25 µg/mL (34). Culturing of strains harboring the temperature-sensitive plasmid pFlp-Ab5 was performed at 30°C, followed by a shift to 37°C to facilitate plasmid curing (64). Induction of *B. cenocepacia* strains was conducted by adding filter-sterilized L-rhamnose monohydrate (Sigma) to LB broth at 0.5% rhamnose or cumate (4-isopropylbenzoic acid, Sigma) dissolved in 100% ethanol and added at concentrations ranging from 2.5 μM to 400 μM. An equivalent volume of solvent (sterile water for rhamnose and ethanol for cumate) was added to the culture media for non-induced controls.

### Recombinant DNA methods

The oligonucleotides utilized in this study are detailed in Table S3. All PCR amplifications for cloning were performed using Q5 DNA polymerase (New England Biolabs, USA), with the inclusion of 2% DMSO for the amplification of *B. cenocepacia* DNA owing to its high GC content. Genomic DNA was isolated using the EZ-10 Spin Column Bacterial Genomic DNA Mini-Preps Kit (Bio Basic, Canada). PCR cleanup and restriction digest purifications were conducted with the Zymoclean Gel DNA Recovery Kit (ZymoResearch, USA). Plasmid DNA was isolated using the QIAprep Spin Miniprep Kit (Qiagen, Germany). Vectors were generated by amplifying regions of interest and then assembling amplicons with the aid of Gibson Assembly (65) using the NEBuilder HiFi DNA Assembly Mix (New England Biolabs). Before Gibson Assembly DNA fragments were evaluated for correctness based on size through agarose gel electrophoresis. Single-nucleotide mutations were generated using Q5 PCR-based site-directed mutagenesis, followed by *DpnI* (New England Biolabs) treatment to remove template DNA. Plasmid mixtures were introduced into chemically competent *E. coli* PIR2 cells through heat shock transformation. The cells were plated on selective media and screened via colony PCR using GoTaq Green Master Mix (Promega, USA). All plasmids utilized in this study were verified by either Sanger sequencing using the Australian Genome Research Facility (Melbourne, Australia) or nanopore whole plasmid sequencing performed by Plasmidsaurus (SNPsaurus LLC, USA). A summary of all plasmids and the primer combinations used for their construction are provided in Table S2. To enhance sharing of resources, plasmids created in this study have been deposited to the Addgene plasmid repository.

### Conjugation of plasmids into *B. cenocepacia*

Plasmids were introduced into *B. cenocepacia* K56-2 strains utilizing triparental mating (34) and *E. coli* PIR2 strains carrying donor plasmids. Conjugations were allowed to proceed for 24 h, then successful transconjugants were selected with Trimethoprim or Tetracycline, and the introduction of plasmids of interest confirmed using PCR-based screening. For the generation of strains containing CTX integration vectors (40), the Tetracycline resistance marker was excised by the introduction of the temperature sensitive flippase vector pFLP-Ab5 (34), with excision confirmed using antibiotic screening and PCR based confirmation. pFLP-Ab5 plasmid curing was undertaken without selection at 37°C.

### Growth conditions and induction assays

sfGFP fluorescence was quantified in *E. coli* and *B. cenocepacia* utilizing a CLARIOstar Plus multimode plate reader (BMG Labtech, Germany) with black, flat-bottom 96-well plates (200 µL per well, *n* = 3). Cultures were inoculated with varying concentrations of 4-isopropylbenzoic acid (cumate, 0–200 µM for *E. coli*; 0–400 µM for *B. cenocepacia*) and incubated at 37°C. Optical density was measured at 600 nm to assess cell density, and GFP fluorescence was quantified with an excitation at 485 nm and emission between 520 and 530 nm. Measurements were taken at 10-min intervals over a 24-h period, with orbital shaking applied before each measurement to ensure uniformity. Background fluorescence and OD values were corrected using blank controls, and fluorescence intensities were normalized to OD₆₀₀ to account for variation in growth rates across conditions.

### Western blotting

Overnight bacterial cultures (1 mL of stationary phase, OD_600nm_ ~1.5) were pelleted then boiled in 1× SDS-PAGE sample buffer (2% SDS, 10% glycerol, 5% 2-mercaptoethanol, 0.004% bromphenol blue, and 75 mM Tris HCl, pH 6.8) for 10 min. Protein lysates were separated on NuPAGE 4%–12% Bis-Tris gradient polyacrylamide gels in MES buffer (Thermo Fisher Scientific). Proteins were transferred to a nitrocellulose membrane using the iBlot 3 Western transfer system (Thermo Fisher Scientific) and blocked for 1 h in 5% skim milk in TBS-T (Tris buffered saline with 0.1% Tween-20). Nitrocellulose membranes were probed with either mouse α-histidine tag (1:1,000; clone: AD1.1.10, Bio-Rad) or mouse α-GFP (1:5,000; IgG1κ clones 7.1 and 13.1, 11814460001, Sigma) overnight at 4°C, washed three times with TBS-T, and then incubated with horseradish peroxidase (HRP) conjugated α-mouse antibody (1:5,000; NEF82200-1EA, PerkinElmer) for 1 h at room temperature. Proteins were detected using Clarity Western ECL Substrate (Bio-Rad) and images obtained using an Amersham imager 800 (GE Healthcare Life Sciences). Membranes were Stripped with Restore Plus Stripping Buffer for 10 min before being re-blocked in 5% skim milk in TBS-T and probed with mouse α-*E. coli* RNA polymerase primary antibody (1:5,000; clone: 4RA2, Neoclone) or mouse α-Actin (1:5,000; clone: BA3R, Thermo Fisher Scientific) followed by HRP conjugated α-mouse and imaged as above. All antibodies were diluted in TBS-T with 1% bovine serum albumin (BSA; Sigma-Aldrich). Western images were saved as enhanced chemiluminescence (ECL) images and as combined colourimetric and ECL images. ECL alone images were used for comparisons to enhance the dynamic range of detection; as a result, backgrounds appear white in the Western blots. All raw, uncropped ECL and combined Western blot images are provided in Fig. S9 to S11.

### Gentamicin protection assays

Gentamicin protection assays were undertaken using the approach of Hamad et al. (47). Briefly, THP-1 cells (ATCC TIB-202) grown in RPMI-1640 culture media (Gibco, Thermo Fisher) supplemented with 10% Fetal Bovine Serum (FBS) at 37°C with 5% CO_2_ were seeded in twenty-four-well plates at a final concentration of 3.0×10^5^ and differentiated with 50 ng/mL Phorbol 12-myristate 13-acetate for 72 h prior to use in infection assays. Overnight cultures of *B. cenocepacia* were standardized to an OD_600_ of 1.0 (~1 × 10^9^ cells per mL) and washed three times with 1 mL of pre-warmed RPMI-1640 with 10% FBS. Bacterial cells were resuspended into 1 mL of fresh RPMI + 10% FBS and 100 µL of 6.0 × 10^5^ cfu/mL, corresponding to a multiplicity of Infection of 2, then added to the differentiated THP-1 cells. To synchronize and enhance bacterial uptake plates were centrifuged for 1 min at 300 × *g* and incubated for 1 h at 37°C under 5% CO_2_. Infected macrophages were washed three times with PBS to remove external bacteria. The media was then replaced with fresh RPMI + 10% FBS containing 50 μg/mL Gentamicin for 30 min, and then the media replaced with RPMI + 10% FBS containing 10 μg/mL Gentamicin. For *in vivo* induction, infected THP 1 cells were maintained in RPMI media containing 10% FBS, 10 μg/mL Gentamicin, and 100 μM cumate following 30 min of 50 μg/mL Gentamicin killing. After a total of 24 h from the initial addition of bacteria, cells were washed once with PBS then lysed with 1% Triton X-100 in PBS. Recovered intracellular bacteria were enumerated by plating serial dilutions on LB agar plates.

### Immunofluorescence microscopy

THP-1 cells were seeded and differentiated on coverslips in 24-well plates as above prior to infection with *B. cenocepacia* strains at an MOI of 2. Twenty-four hours post-infection, the culture media was removed and cells washed with PBS then fixed by the addition of 4% pre-chilled paraformaldehyde and incubated at room temperature for 15 min. Fixed cells were washed with PBS five times and then permeabilized in PBS containing 1% BSA and 0.2% Triton X-100 for 1 h, before blocking in PBS containing 1% BSA and 0.1% Tween-20 for 1 h. Cells were stained with α-*Burkholderia* polyclonal rabbit serum (1:500 in PBS with 1% BSA—generated from lysates of *B. cenocepacia K56-2* by the Walter and Eliza Hall Institute antibody facility) for 1 h. Unbound antibodies were removed by washing with PBS containing 1% BSA and 0.1% Tween-20 three times for 10 min and coverslips then incubated with α-rabbit Alexa Fluor 568 secondary antibody (1:2,000 in PBS containing 1% BSA and 0.1% Tween-20) for 1 h in the dark. Unbound secondary antibody was removed by washing three times with PBS containing 1% BSA and 0.1% Tween-20 three times for 10 min before the addition of DNA and actin stains Hoechst 33,342 (0.1 µg/mL, Abcam) and SiR-Actin (100 nM, Spirochrome) for 20 min in PBS containing 1% BSA. Coverslips were then washed five times with PBS containing 1% BSA and 0.1% Tween-20 before being mounted using ProLong Gold Antifade (Invitrogen) on a glass slide and allowed to cure overnight and subsequently sealed with nail polish. Imaging was conducted utilizing an LSM980 confocal microscope (Zeiss, Germany) using the smart setup acquisition mode and an oil immersion 63× objective. Z-stack images were reconstructed through orthogonal projection, and visualization was performed using Fiji software (66) and three independent infection assays undertaken. Image processing was restricted to linear modifications of brightness and contrast, uniformly applied to all samples.

### Proteomic sample preparation

*B. cenocepacia* cultures for proteomic analysis were incubated overnight with or without inducers (Cumate or Rhamnose), under shaking conditions at 180 rpm. Cultures were adjusted to an OD₆₀₀ of 1.0, harvested by centrifugation at 10,000 × *g* for 10 min at 4°C, washed three times with ice-cold PBS, and snap-frozen at −80°C until further processing. Frozen whole-cell samples were solubilized in 4% SDS, 100 mM Tris pH 8.5 by boiling for 10 min at 95°C, and then protein concentrations assessed by bicinchoninic acid protein assays (Thermo Fisher Scientific). One hundred micrograms of each biological replicate/sample was prepared for digestion using S-traps mini columns (Protifi, USA) according to the manufacturer’s instructions. Briefly, samples were reduced with 10 mM Dithiothreitol for 10 min at 95°C and then alkylated with 50 mM Iodoacetamide in the dark for 1 h. Samples were acidified to 1.2% phosphoric acid and diluted with seven volumes of S-trap wash buffer (90% methanol, 100 mM Tetraethylammonium bromide pH 7.1) before being loaded onto S-traps and washed three times with S-trap wash buffer. Samples were then digested with 2 μg of SoluTrypsin (Sigma) overnight at 37°C before being collected by centrifugation with washes of 100 mM Tetraethylammonium bromide, followed by 0.2% formic acid followed by 0.2% formic acid/ 50% acetonitrile. Samples were dried down and further cleaned up using C_18_ Stage (67, 68) tips to ensure the removal of any particulate matter.

### Data-dependent acquisition LC-MS analysis

Combined Glycopeptide and proteomic analysis were undertaken using DDA-based analysis. Samples were re-suspended in Buffer A* (2% acetonitrile, 0.1% trifluoroacetic acid) and separated using a two-column chromatography setup composed of a PepMap100 C_18_ 20-mm by 75-μm trap (Thermo Fisher Scientific) and a PepMap C_18_ 500-mm by 75-μm analytical column (Thermo Fisher Scientific) using a Dionex Ultimate 3000 UPLC (Thermo Fisher Scientific). Samples were concentrated onto the trap column at 5 μL/min for 6 min with Buffer A (0.1% formic acid, 2% DMSO) and then infused into an Orbitrap Fusion Lumos (Thermo Fisher Scientific) at 300 nL/min via the analytical column. Peptides were separated by altering the buffer composition from 3% Buffer B (0.1% formic acid, 77.9% acetonitrile, 2% DMSO) to 28% B over 85 min, then from 23% B to 40% B over 4 min, and then from 40% B to 80% B over 3 min. The composition was held at 80% B for 2 min before being returned to 3% B for 10 min. The Orbitrap Lumos Mass Spectrometer was operated in a data-dependent mode automatically switching between the acquisition of a single Orbitrap MS scan (300–2,000 *m*/*z*, maximal injection time of 25 ms, an Automated Gain Control [AGC] set to a maximum of 300% and a resolution of 60k) and 3 s of Orbitrap MS/MS HCD scans of precursors (NCE of 35%, a maximal injection time of 54 ms, a AGC of 100% and a resolution of 15). HCD scans containing HexNAc-associated oxonium ions (204.0867, 138.0545, and 366.1396 *m*/*z*) triggered two additional product-dependent MS/MS scans (69) of potential glycopeptides; an Orbitrap EThcD scan (NCE 15%, maximal injection time of 150 ms, AGC set to a maximum of 250% ions with a resolution of 30k using the extended mass range setting to improve the detection of high mass glycopeptide fragment ions [70]) and a stepped collision energy HCD scan (using NCE 35% with 5% Stepping, maximal injection time of 150 ms, an AGC set to a maximum of 250% ions and a resolution of 30k).

### Data-independent acquisition LC-MS analysis

Total proteome analysis was undertaken using DIA-based analysis. C_18_ enriched proteome samples were re-suspended in Buffer A* and separated using a two-column chromatography setup composed of a PepMap100 C_18_ 20-mm by 75-μm trap (Thermo Fisher Scientific) and a PepMap C_18_ 500-mm by 75-μm analytical column (Thermo Fisher Scientific) using a Dionex Ultimate 3000 UPLC (Thermo Fisher Scientific). Samples were concentrated onto the trap column at 5 μL/min for 6 min with Buffer A (0.1% formic acid, 2% DMSO) and then infused into an Orbitrap Eclipse (Thermo Fisher Scientific) at 300 nL/min via the analytical column. Peptides were separated by altering the buffer composition from 3% Buffer B (0.1% formic acid, 77.9% acetonitrile, 2% DMSO) to 28% B over 70 min, from 23% B to 40% B over 4 min, and then from 40% B to 80% B over 3 min. The composition was held at 80% B for 2 min before being returned to 3% B for 10 min. The Orbitrap Eclipse was operated in a data-independent mode automatically switching between the acquisition of a single Orbitrap MS1 event (120k resolution, AGC set to a maximum of 100%, 350–1,400 *m*/*z*) and 50 MS2 scans (NCE 30%, 30k resolution, AGC set to a maximum of 1000%, 200–2,000 *m*/*z* and a maximal injection time of 55 ms) of a width of 13.7 *m*/*z* collected over the mass range of 361 to 1,033 *m*/*z*.

### DDA-based proteomic analysis

DDA data sets were analyzed using MSFragger (version 22.0) (71–73). Samples were searched with a Tryptic specificity, allowing a maximum of two missed cleavage events and Carbamidomethyl set as a fixed modification of Cysteine while oxidation of Methionine allowed as a variable modification. The *Burkholderia* glycans HexHexNAc_2_ (elemental composition: C_22_O_15_H_36_N_2_, mass: 568.2115 Da) and Suc-HexHexNAc_2_ (elemental composition: C_26_O_18_H_40_N_2_, mass: 668.2276 Da) were included as variable modifications at Serine in line with the strong preference for PglL glycosylation at Serine residues (60, 61). The glycan fragment ions were defined as 204.0866, 186.0760 168.0655, 366.1395, 144.0656, 138.0550, 466.1555, and 407.1594. A maximum mass precursor tolerance of 20 ppm was allowed at both the MS1 and MS2 levels. Samples were searched against the *B. cenocepacia* reference proteome J2315 (Uniprot accession: UP000001035, 6,993 proteins) (74) supplemented with the proteins RhaS (HTH-type transcriptional activator RhaS, Uniprot accession: P09377), RhaR (HTH-type transcriptional activator RhaR, Uniprot accession: P09378), TmpR (Dihydrofolate reductase resistance marker, Uniprot accession: P00384), and CymR (HTH-type transcriptional regulator CymR, Uniprot accession: O33453). MS1-based quantitation was undertaken using MaxLFQ (75) with matching between runs enabled. Analysis of protein abundances was undertaken using Perseus (76) with data visualization and was undertaken using ggplot2 (77) in R.

### DIA-based proteomic analysis

DIA data were searched with MSFragger (version 22.0) (71–73) using the “DIA_SpecLib_Quant” workflow utilizing DIA-NN (78). Samples were searched with a Tryptic specificity, allowing a maximum of two missed cleavage events and Carbamidomethyl set as a fixed modification of Cysteine while oxidation of Methionine allowed as a variable modification. A maximum mass precursor tolerance of 20 ppm was allowed at both the MS1 and MS2 levels. Samples were searched against the *B. cenocepacia* reference proteome J2315 (Uniprot accession: UP000001035, 6,993 proteins) (74) supplemented with the proteins RhaS (HTH-type transcriptional activator RhaS, Uniprot accession: P09377), RhaR (HTH-type transcriptional activator RhaR, Uniprot accession: P09378), TmpR (Dihydrofolate reductase resistance marker, Uniprot accession: P00384), and CymR (HTH-type transcriptional regulator CymR, Uniprot accession: O33453). DIA-NN-based quantitation was used for statistical analysis of protein abundances and analyzed in Perseus (76). Missing values were imputed based on the total observed protein intensities with a range of 0.3 σ and a downshift of 2.5 σ with biological replicates grouped together to allow comparison by student *t*-tests. Proteins were considered altered if they satisfied both *P*-value and fold change alterations as defined by the function *y* = *c*/(*x* − x0) where *c* = curvature and x0 = minimum fold change with were x0 = 1 and *c* = 2 (79). Principal Component Analysis (PCA) and data visualization were undertaken using ggplot2 (77) in R.

## Data Availability

All raw mass spectrometry data, analysis outputs, and scripts have been deposited in the PRIDE ProteomeXchange repository. Accession numbers (PXD070989, PXD070223, PXD075637, and PXD070285) and experiment details are provided in Table S4. Plasmids have been deposited in Addgene under accession numbers 253929 to 253933.

## SUPPLEMENTAL MATERIAL

Supplemental material aem.00532-26-s0001.docx Tables S1 to S4, legends for Tables S5 to S11, and Fig. S1 to S11.

Table S5 aem.00532-26-s0002.xlsx DDA PSM summary of *B. cenocepacia* K56-2 Δ*pglL-bcal1086*-his containing pCumate-sfGFP and pCumate-PglL*_Bc_*-his with and without induction.

Table S6 aem.00532-26-s0003.xlsx DDA peptide and protein identification tables of *B. cenocepacia* K56-2 Δ*pglL-bcal1086*-his containing pCumate-sfGFP and pCumate-PglL*_Bc_*-his with and without induction.

Table S7 aem.00532-26-s0004.xlsx DDA peptide and protein identification tables of *B. cenocepacia* K56-2 Δ*pglL-bcal1086*-his containing pCumate_GV_-PglL*_Bc_*-his and pCumate-PglL*_Bc_*-his with and without induction.

Table S8 aem.00532-26-s0005.xlsx DDA PSM summary of *B. cenocepacia* K56-2 Δ*pglL-bcal1086*-his containing pCumateGV-PglL*_Bc_*-his and pCumate-PglL*_Bc_*-his with and without induction.

Table S9 aem.00532-26-s0006.xlsx DIA proteomic analysis of *B. cenocepacia* K56-2 harboring pCumate-sfGFP, pSCrhaB2, and pBBR1-EV.

Table S10 aem.00532-26-s0007.xlsx DDA PSM summary of *B. cenocepacia* K56-2 Δ*amrAB*Δ*pglL* and Δ*amrAB*Δ*pglL* containing chromosomally integrated CTX-CymR_GV_-PglL*_Bc_*-his with and without induction.

Table S11 aem.00532-26-s0008.xlsx DDA peptide and protein identification tables of *B. cenocepacia* K56-2 Δ*amrAB*Δ*pglL* and Δ*amrAB*Δ*pglL* containing chromosomally integrated CTX-CymR_GV_-PglL*_Bc_*-his with and without induction.

ASM does not own the copyrights to Supplemental Material that may be linked to, or accessed through, an article. The authors have granted ASM a non-exclusive, world-wide license to publish the Supplemental Material files. Please contact the corresponding author directly for reuse.
