# Supplementary material for: Optimized cumate toolkit for tunable protein expression during in vitro and in vivo studies of Burkholderia cenocepacia
Source: Appl Environ Microbiol. 2026 Jun 9;92(7):e00532-26. doi: 10.1128/aem.00532-26 (PMC13390352; doi:10.1128/aem.00532-26)
Supplement: Supplemental material — Tables S1 to S4, legends for Tables S5 to S11, and Fig. S1 to S11. [file aem.00532-26-s0001.docx]

**Optimised Cumate toolkit for tuneable protein expression during *in vitro* and *in vivo* studies of *Burkholderia* *cenocepacia***

Hamza Tahir^1^, Kristian I. Karlic^1^, Godfrey Mwiti^1^, and Nichollas E. Scott^1^

^1^Department of Microbiology and Immunology, University of Melbourne, Peter Doherty Institute for Infection and Immunity, Melbourne, Victoria, Australia

**Table of Content**

| **Title** | **Page** |
| --- | --- |
| Supplementary Table 1: Strain List | 2 |
| Supplementary Table 2: Plasmid List | 3-4 |
| Supplementary Table 3: Primer List | 5-7 |
| Supplementary Table 4: Proteomic datasets | 8 |
| Supplementary Tables 5-11: Proteomic Supplementary Table Descriptions | 9-10 |
| Supplementary Figures 1-11 | 11-20 |
| References | 21 |

| **Strain** | **Description** | **Source** |
| --- | --- | --- |
| ***E. coli*** | | |
| DH5α | F^−^ Φ80*lac*ZΔM15 Δ(*lac*ZYA-*argF*) U169 *rec*A1 *end*A1 *hsd*R17(r_K_^–^, m_K_^+^) *pho*A *sup*E44 *thi*-1 *gyr*A96 *rel*A1 λ^–^ | Invitrogen |
| PIR2 | F^−^ ∆*lac*169 *rpo*S(am) *rob*A1 *cre*C510 *hsd*R514 *end*A *rec*A1 *uidA*(∆*Mlu*I)::*pir* | Thermo Scientific |
| ***Burkholderia Strains*** | | |
| K56-2 WT | CF clinical isolate of the ET12 lineage, closely related to *B. cenocepacia* J2315 | Canadian *B. cepacia* research and referral repository [1] |
| K56-2 Δ*pglL* | *B.* cenocepacia K56-2 lacking *PglL (BCAL0960)* | [2] |
| K56-2 ∆*pglL* BCAL1086-his | *B.* cenocepacia K56-2 lacking *PglL (BCAL0960)* and containing chromosomally his tagged BCAL1086 | [2] |
| K56-2 MH1K | *B.* cenocepacia K56-2 lacking *amrAB-oprA* (*BCAL1674-BCAL1676*)*.* Gentamicin sensitive strain. | [3] |
| K56-2 MH1K CTX::sfGFP | *B.* cenocepacia K56-2 lacking *amrAB-oprA* (*BCAL1674-BCAL1676).* Contains chromosomally integrated CTX with sfGFP under P_CymRC_/CymR_GV_ control. Gentamicin sensitive strain. | This study |
| K56-2 Δ*amr*AB | *B.* cenocepacia K56-2 lacking *amr*AB (*BCAL1674-BCAL1675*)*.* Gentamicin sensitive strain created using plasmid pMH447. | This study |
| K56-2 Δ*amr*AB Δ*pglL* | *B.* cenocepacia K56-2 lacking *pglL* (*BCAL0960*) and *amr*AB (*BCAL1674-BCAL1675).* Gentamicin sensitive strain created using plasmid pMH447 from strain K56-2 Δ*pglL.* | This study |
| K56-2 Δ*amr*AB Δ*pgl*L CTX::PglL_Bc_-his | *B.* cenocepacia K56-2 lacking *PglL* (*BCAL0960*) and *amr*AB (*BCAL1674-BCAL1675)*. Contains chromosomally integrated CTX with PglL_Bc_-his under P_CymRC_/CymR_GV_ control. Gentamicin sensitive strain | This study |
| *B. thailandensis* E264 | Reference *B. thailandensis* strain isolated from Rice-field soil sample in Thailand*.* Obtained from Deborah Yoder-Himes; University of Louisville | [4] |

**Supplementary Document Table 1: Strain list**

**Supplementary Document Table 2: Plasmid list**

| **Plasmid** | **Description** | **Source** |
| --- | --- | --- |
| pUS250-sfGFP | Derivative of pUS250 (Addgene plasmid # 198322) containing the cumate-inducible system of *Pseudomonas putida* (P_Cym_/CymR) regulating the expression of superfolder green fluorescent protein (sfGFP). Kan^R^ | Unpublished plasmid, Coleman lab |
| pMLBAD | *ori*_pBBR1_, *araC* P_BAD_, *mob^+^*, Tmp^R^. Addgene plasmid # 32055 | [5] |
| pMH447 | pGPI-SceI with fragments flanking ∆*amrAB* (BCAL1674–BCAL1675) | [6] |
| pMH447-S7-pglL-his | pMH447 vector backbone, containing His- tagged PglL from *B. cenocepacia* K56-2 under the S7 promoter. Tmp^R^ | [2] |
| pRK2013 | Helper plasmid for conjugation*, ori*_colE1_, RK2 derivative, *mob*^+^, *tra*^+^, Kan^R^ | [7] |
| pFlp-Ab5 | pFlpe4-derived plasmid, rhamnose-inducible *flp* (flippase), Tet^R^, temperature-sensitive | [8] |
| pSCrhaB2 | *ori*_pBBR1_, *rhaR*, *rhaS*, P*_rhaB_*, *mob^+^,* Tmp^R.^  Addgene Plasmid #113634 | [9] |
| pKCyR5 | Broad-Host range expression vector, RK2 and pUC origin, containing P_CymRC_/CymR_AM_ controlling mRFP. Gent^R^. Addgene Plasmid #149463 | [10] |
| pAH-CTX1-Rha | Mini-CTX1 integration vector containing rhaR rhaS P_rhaB_, ori_pMB1_, intφCTX, oriT, Tet^R^ | [11] |
| pCumate-sfGFP  (Addgene ID 253929) | *ori*_pBBR1_, *mob^+^*, plasmid derived from pMLBAD containing the cumate-inducible system P_Cym_/CymR controlling *sfGFP.* Generated using Gibson assembly with PCR products generated with Nsco_1094 / Nsco_1095 from pUS250-sfGFP and Nsco_1092 / Nsco_1093 from pMLBAD. Tmp^R^ | This study |
| pCumate-PgIL_Bc_-his | pCumate vector containing *pgl*L *(BCAL0960)* from *B. cenocepacia*. Nsco_1200 / Nsco_1201 from pMH447-S7-pglL-his and Nsco_1202 / Nsco_1203 from pCumate-sfGFP. Tmp^R^ | This study |
| pCumate^S110G^-sfGFP | pCumate-sfGFP derivative containing Serine^110^ to Glycine^110^ substitution in CymR. Nsco_1433 / Nsco_1093 and Nsco_1434 / Nsco_1095 using pCumate-sfGFP. Tmp^R^ | This study |
| pCumate^A171V^-sfGFP | pCumate-sfGFP derivative containing Alanine ^171^ to Valine^171^ substitution in CymR. Nsco_1435 / Nsco_1093 and Nsco_1436 / Nsco_1095 using pCumate-sfGFP. Tmp^R^ | This study |
| pCumate^S110G A171V^-sfGFP | pCumate^S110G^-sfGFP derivative. Double substitution plasmid of Serine^110^ to Glycine^110^ and Alanine^171^ to Valine^171^. Nsco_1435 / Nsco_1434 and Nsco_1433 / Nsco_1436 using pCumate-sfGFP. Tmp^R^ | This study |
| pCumate_AM_-sfGFP | pCumate-sfGFP derivative with P_CymRC_/CymR_AM_ circuit from pKCyR5 used to replace P_Cym_/CymR. Nsco_1501 / Nsco_1582 from pCumate-sfGFP and Nsco_1583 / Nsco_1495 from pKCyR5. Tmp^R^ | This study |
| pCumate_AM_^-G110S^-sfGFP | pCumate_AM_-sfGFP derivative containing Glycine^110^ to Serine^110^ substitution in CymR. Nsco_1093 / Nsco_1630 and Nsco_1095 / Nsco_1631 from pCumate^Am^-sfGFP. Tmp^R^ | This study |
| pCumate_AM_^-V171A^-sfGFP | pCumate_AM_-sfGFP derivative containing Valine ^110^ to Alanine ^110^ substitution in CymR Nsco_1093 / Nsco_1632 and Nsco_1095 / Nsco_1633 from pCumate_Am_-sfGFP. Tmp^R^ | This study |
| pCumate_GV_ -sfGFP  (Addgene ID 253930) | pCumate_AM_^-G110S^-sfGFP derivative containing double substitution of Glycine^110^ to Serine^110^ and Valine^171^ to Alanine ^171^ in CymR. Nsco_1630 / Nsco_1631 and Nsco_1631 / Nsco_1632 from pCumate^Am^-sfGFP, Tmp^R^ | This study |
| pCumate_GV_-PglL*_Bc_*-his | pCumate_GV_-sfGFP derivative containing *B. cenocepacia* PglL-his. Nsco_1200 / Nsco_1201 from pCumate-PglL and Nsco_1202 / Nsco_1203 from pCumate^GV^-sfGFP. Tmp^R^ | This study |
| pCTX-CymR_GV_-sfGFP  (Addgene ID 253933) | CTX based integration vector containing P_CymRC_/CymR_GV_. Derived from pAH-CTX-Rha replacing the rhamnose induction circuit with P_CymRC_/CymR_GV_ controlling sfGFP from pCumate_GV_-sfGFP. Nsco_1813 / Nsco_1814 from pAH-CTX1-rha and Nsco_1815 / Nsco_1816 from pCumate^GV^ -sfGFP. Tet^R^ | This study |
| pCTX-CymR_GV_-PglL_Bc_-his | CTX based integration vector containing P_CymRC_/CymR_GV_. Derived from pCTX-CymR_GV_-sfGFP replacing the sfGFP with PglL_Bc-_his from pCumate_GV_-PglL*_Bc_*-his. Nsco_1813 / Nsco_1890 from pCTX-CymR^GV^-sfGFP and Nsco_1816 / Nsco_1889 from pCumate^GV^-PglL*_Bc_*-his. Tet^R^ | This study |
| pCumate_GV_ -MCS  (Addgene ID 253931) | pCumate_GV_-sfGFP derivative containing only a multiple cloning site (MCS). Generated from pCumate_GV_-sfGFP using Nsco_1930 / Nsco_1931. Tmp^R^ | This study |
| pBBR1-EV | pBBR1 empty vector backbone amplified from pCTX-CymR_GV_-sfGFP using Nsco_1931 / Nsco_1932. Tmp^R^ | This study |
| pCTX-CymR_GV_-MCS  (Addgene ID 253932) | CTX based integration vector containing P_CymRC_/CymR_GV_ upstream of a multiple cloning site. Generated using pCTX-CymR_GV_-sfGFP and Nsco_1930 / Nsco_1934. Tet^R^ | This study |

Kan^R^, Kanamycin resistance; Tet^R^, Tetracycline resistance; Amp^R^, Ampicillin resistance; Tmp^R^, trimethoprim resistance; Gent^R.^, Gentamicin resistance.

**Supplementary Document Table 3: Primer list**

| **Primer** | **Sequence** | **Description / Purpose** |
| --- | --- | --- |
| Nsco_1092 | CTGAAATTTGCTTCGGGGTCATTATAGggtctgataaaacagaatttgcctgg | Forward primer to amplify pMLBAD backbone to create pCumate-sfGFP |
| Nsco_1093 | CCTTTTTCTTTAAAACCGAAAAGATTACCgatgggagatcctaagatatcgc | Reverse primer to amplify pMLBAD backbone to create pCumate-sfGFP |
| Nsco_1094 | ccaggcaaattctgttttatcagaccCTATAATGACCCCGAAGCAAATTTCAG | Forward primer to amplify sfGFP under P_Cym_/CymR from pUS250-sfGFP to create pCumate-sfGFP |
| Nsco_1095 | gcgatatcttaggatctcccatcGGTAATCTTTTCGGTTTTAAAGAAAAAGG | Reverse primer to amplify sfGFP under P_Cym_/CymR from pUS250-sfGFP to create pCumate-sfGFP |
| Nsco_1200 | gagaaatcaaattaaggaggtaagataatgccttctactttttcccgttcg | Forward primer to amplify *B. cenocepacia* pgIL-his from pMH447-S7-PglL-his to insert into pCumate-sfGFP |
| Nsco_1201 | ATATCGATAAGCTTGCTAGCATGCTagacccAAACCCGGGtcaG | Reverse primer to amplify *B. cenocepacia* pgIL-his from pMH447-S7-PglL-his to insert into pCumate-sfGFP |
| Nsco_1202 | CCACCACtgaCCCGGGTTTgggtctAGCATGCTAGCAAGCTTATCGA | Forward primer to amplify pCumate-sfGFP backbone to insert pgIL_Bc_-his |
| Nsco_1203 | cgaacgggaaaaagtagaaggcattatcttacctccttaatttgatttctc | Reverse primer to amplify pCumate-sfGFP backbone to insert pgIL_Bc_-his |
| Nsco_1256 | AGATCTgccatgagaccCAA | Forward screening primer for pCumate-sfGFP |
| Nsco_1257 | TCCATAAAACCGCCCAGTCT | Reverse screening primer for pCumate-sfGFP |
| Nsco_1308 | gccttgaccgaaacggaggaat | PBBR1 backbone plasmid for screening plasmid pCumate-sfGFP along with Nsco_1473. |
| Nsco_1433 | GACGATGACTTCTCTATCGGCCTTGATTTGATTGTGGCTGCCG | Forward primer to substitute Serine to Glycine at position 110 within CymR of pCumate-sfGFP |
| Nsco_1434 | CGGCAGCCACAATCAAATCAAGGCCGATAGAGAAGTCATCGTC | Reverse primer to substitute Serine to Glycine at position 110 within CymR of pCumate-sfGFP |
| Nsco_1435 | AATTCGGTGCGTGGACTTGTTGTTCGTAGCCTATGGCAGAAGG | Forward primer to substitute Alanine to Valine at position 171 within CymR of pCumate-sfGFP |
| Nsco_1436 | CCTTCTGCCATAGGCTACGAACAACAAGTCCACGCACCGAATT | Reverse primer to substitute Alanine to Valine at position 171 within CymR of pCumate-sfGFP |
| Nsco_1473 | TTCGGTGATCTGTTCGTAAAGC | Primer to screen for CymR in pCumate-sfGFP construct to be used in combination with PBBR1 backbone primer Nsco_1308. |
| Nsco_1499 | CTGTATAATAGATTCAACAAACAGAC | Reverse primer to screen P_CymRC_/CymR_GV_ promoter in pCumate^AM^-sfGFP using Nsco_1502 |
| Nsco_1495 | CTCGAGTACTGAGCTCATATGctgtttcgtcctcacggactc | Forward primer to amplify P_CymRC_/CymR_AM_ from plasmid pKCyR5 |
| Nsco_1501 | gagtccgtgaggacgaaacagCATATGAGCTCAGTACTCGAGGATCC | Reverse primer to amplify the pMLBAD vector backbone |
| Nsco_1502 | cccagaatgttaccatcctctt | Forward screening primer for pCumate^AM^-sfGFP |
| Nsco_1582 | atgcaaaattcaaacgttgatcTTGCTTCGGGGTCATTATAGggtc | Forward screening primer for pCumate-sfGFP, use in combination with Nsco_1501. |
| Nsco_1583 | ccCTATAATGACCCCGAAGCAAgatcaacgtttgaattttgcataacgt | Reverse primer to amplify P_CymRC_/CymR_AM_ from pKCyR5plasmid, use with Nsco_1495. |
| Nsco_1586 | AGATCTgccatgagaccCAA | Screening primer for pCumate backbone. Use with Nsco_1933. |
| Nsco_1630 | ggatgatgattttagcatcAgcctggatctgattgttgcagca | Forward primer to substitute Glycine to Serine at position 110 within CymR of pCumate^AM^-sfGFP. Use with Nsco_1093 |
| Nsco_1631 | tgctgcaacaatcagatccaggcTgatgctaaaatcatcatcc | Reverse primer to substitute Glycine to Serine at position 110 within CymR of pCumate_AM_-sfGFP. Use with Nsco_1095 |
| Nsco_1632 | aacagcgttcgtggtctggcagttcgtagcctgtggcagaaag | Forward primer to substitute Valine to Alanine at position 171 within CymR of pCumate_AM_-sfGFP.  Use with Nsco_1093. |
| Nsco_1633 | ctttctgccacaggctacgaactgccagaccacgaacgctgtt | Reverse primer to substitute Valine to Alanine at position 171 within CymR of pCumate_AM_-sfGFP. Use with Nsco_1095 |
| Nsco_1634 | gttatgcaggttttcgtattg | Sanger sequencing primer for mutant CymR plasmids. |
| Nsco_1813 | atgcaaaattcaaacgttgatcttccattgagtaagtttttaagcacatcagct | Forward primer to amplify CTX1 backbone from pAH-CTX1-Rha. Use with Nsco_1814. |
| Nsco_1814 | ggacgagctgtataagtaatgagcgcgggtggatgacCTTTTGAA | Reverse primer to amplify CTX1 backbone from pAH-CTX1-Rha. Use with Nsco_1813. |
| Nsco_1815 | tcaaaaggtcatccacccgcgctcattacttatacagctcgtccataccgtgg | Forward primer to amplify P_CymRC_/CymR_GV_-sfGFP from pCumate_GV_ -sfGFP for cloning into pAH-CTX1 backbone. Use with Nsco_1816. |
| Nsco_1816 | cttaaaaacttactcaatggaagatcaacgtttgaattttgcataacgt | Reverse primer to amplify P_CymRC_/CymR_GV_-sfGFP from pCumate_GV_ -sfGFP for cloning into CTX1 backbone. Use with Nsco_1815. |
| Nsco_1817 | agaacgtgcaatggaaaccc | Forward screening primer for  P_CymRC_/CymR_GV_-sfGFP intergration into CTX1 backbone. Use with Nsco_1818. |
| Nsco_1818 | cgggcaggataggtgaagta | Reverse screening primer for  P_CymRC_/CymR_GV_-sfGFP intergration into CTX1 backbone. Use with Nsco_1817. |
| Nsco_1819 | aaaatggacagtagggccca | Forward screening, binding backbone of CTX1. Use with Nsco_1820 |
| Nsco_1820 | gctactacaaaactcgcgca | Reverse screening primer binding within sfGFP. Use with Nsco_1819 |
| Nsco_1884 | gtgctattacgcacacgttc | Reverse screening primer for P_CymRC_/CymR_GV_. use with Nsco_1634 |
| Nsco_1889 | attcaaaaggtcatccacccgcgcTTGCTAGCATGCTagacccAaac | Forward primer to amplify PglL_Bc_ from pCumate^GV^-PglL*_Bc_*-his to construct pCTX-CymR^GV^-PglLBc-his. Use with Nsco_1816. |
| Nsco_1890 | gTTTgggtctAGCATGCTAGCAAgcgcgggtggatgaccttttgaa | Forward primer to amplify pCTX-CymR^GV^-sfGFP backbone to allow insertion of PglL_Bc_ from pCumate^GV^-PglL*_Bc_*-his to construct pCTX-CymRGV-PglLBc-his. Use with Nsco_1813. |
| Nsco_1891 | agatcgcgaacaactcgcac | Reverse screening primer for PglL_Bc_-his insertion into pCTX-CymR^GV^-sfGFP. Use with Nsco_1819 to screen vector or Nsco_1904 to screen integration. |
| Nsco_1904 | GTTTCCTCAACGACCTGCAG | Forward primer used to screen integration using Mini-CTX1. Use with Nsco_1891. |
| Nsco_1930 | tAgCCATGGTCCCGGGTTCTAGAgatttctctagtagctagAAGCGGC | Forward primer to add MCS in pCymR_GV_-sfGFP to generate pCymR_GV_-MCS. Use with Nsco_1931. |
| Nsco_1931 | TCTAGAACCCGGGACCATGGcTaACTAGTAGCGGCCGCTGCAGCATGCT | Reverse primer to add MCS in pCymR_GV_-sfGFP to generate pCymR_GV_-MCS. Use with Nsco_1930. |
| Nsco_1932 | tAgCCATGGTCCCGGGTTCTAGAacagaatttgcctggcggcagtag | Forward primer to generate pBBR1_EV. Use with Nsco_1931. |
| Nsco_1933 | tgcttcgcaacgttcaaatc | Screening primer to use in combination with Nsco 1586 to screen pBBR1-EV backbone. |
| Nsco_1934 | TCTAGAACCCGGGACCATGGcTacgcgggtggatgacCTTTTGAATGA | Reverse primer to generate pCTX-CymR_GV_-MCS from pCTX-CymR_GV_-sfGFP plasmid. Use with Nsco_1930. |

**Supplementary Table 4: Proteomic Dataset**

| Pride accession number (Review login details) | MS instrument | Number of Biological groups, replicates and total datafiles | Description of dataset |
| --- | --- | --- | --- |
| PXD070989 | Orbitrap Fusion Lumos | 4 biological groups, 4 replicates total of 16 datafiles | DDA experiment assessing glycosylation and expression of PglL protein from pCumate-PglL within K56-2 Δ*pglL-bcal1086-*his at stationary phase grown in LB with induced and uninduced condition. pCumate-sfGFP plasmid used as expression control vector. |
| PXD070223 | Orbitrap Fusion Lumos | 4 biological groups, 4 replicates total of 16 datafiles | DDA experiment assessing glycosylation and expression of PglL protein from pCumate-PglL and pCumate _GV_^-^PglL within K56-2 Δ*pglL-bcal1086-*his at stationary phase grown in LB with induced and uninduced condition. |
| PXD075637 | Orbitrap Eclipse | 7 biological groups, 4 replicates total of 28 datafiles | DIA experiment assessing proteomic alteration in response to cumate (100 μM) and L-rhamnose (0.5%) within *B. cenocepacia* K56-2 at stationary phase grown in LB. |
| PXD070285 | Orbitrap Fusion Lumos | 3 biological groups, 4 replicates total of 12 datafiles | DDA experiment assessing glycosylation and expression of PglL protein from chromosomally complemented CTX-CymR_GV_^-^PglL within K56-2 Δa*mr*ABΔ*pgl*L in induced and uninduced conditions (100 μM cumate) at stationary phase grown in LB. |

**Supplementary Table 5. DDA PSM summary of *B. cenocepacia* K56-2 Δ*pglL-bcal1086-*his containing pCumate-sfGFP and pCumate-PglL*_Bc_-*his with and without induction*.*** The MSfragger PSMs search summary for the proteome analysis of *B. cenocepacia* Δ*pglL-bcal1086-*his containing pCumate-sfGFP and pCumate-PglL*_Bc_-his* strains are provided. For each modification type identified within the proteome the total number of PSMs assigned are tabulated.

**Supplementary Table 6. DDA peptide and protein identification tables of *B. cenocepacia* K56-2 Δ*pglL-bcal1086-*his containing pCumate-sfGFP and pCumate-PglL*_Bc_*-his with and without induction.** MSfragger search results for the protein analysis of four biological replicates of strains *B. cenocepacia* K56-2 Δ*pglL-bcal1086-*his containing pCumate-sfGFP and pCumate-PglL*_Bc_-his* without and with induction with 100 μM cumate. For each peptide and protein, the identification information (including modification assignments) and quantitative information including ion intensities as well as if the assignment was based on matching or by MS/MS identifications across the biological replicates are provided.

**Supplementary Table 7. DDA peptide and protein identification tables of *B. cenocepacia* K56-2 Δ*pglL-bcal1086-*his containing pCumate_GV_-PglL*_Bc_*-his and pCumate-PglL*_Bc_*-his with and without induction.** MSfragger search results for the protein analysis of four biological replicates of strains *B. cenocepacia* K56-2 Δ*pglL-bcal1086-*his containing pCumate_GV_-PglL*_Bc_*-his and pCumate-PglL*_Bc_*-his without and with induction with 100 μM cumate. For each peptide and protein, the identification information (including modification assignments) and quantitative information including ion intensities as well as if the assignment was based on matching or by MS/MS identifications across the biological replicates are provided.

**Supplementary Table 8. DDA PSM summary of *B. cenocepacia* K56-2 Δ*pglL-bcal1086-*his containing pCumate_GV_-PglL*_Bc_*-his and pCumate-PglL*_Bc_*-his with and without induction*.*** The MSfragger PSMs search summary for the proteome analysis of *B. cenocepacia* Δ*pglL-bcal1086-*his containing pCumate_GV_-PglL*_Bc_*-his and pCumate-PglL*_Bc_*-his strains are provided. For each modification type identified within the proteome the total number of PSMs assigned are tabulated.

**Supplementary Table 9. DIA proteomic analysis of B. cenocepacia K56-2 harbouring pCumate-sfGFP (±100 μM cumate), pSCrhaB2 (±0.5% L-rhamnose), and pBBR1-EV (uninduced or induced with 100 μM cumate or 0.5% L-rhamnose).** The Perseus processed MSfragger-DIA searches results for protein level analysis of biological replicates (n=4) grown +/- the addition of Rhamnose/Cumate. Imputed Perseus processed data with statistical analysis is provided. For each identified protein, the log_2_ LFQ protein values, T-‑test information including the -log_10_(*p*-value), difference in the mean between the groups and if the resulting *p*-values are below the multiple hypothesis corrected *p*‑values are provided. Categorical information associated with protein accessions, gene name, and GO terms has also been provided.

**Supplementary Table 10. DDA PSM summary of *B. cenocepacia* K56-2 Δ*amrAB*Δ*pglL* and Δ*amrAB*Δ*pglL* containing chromosomally integrated CTX-CymR_GV_-PglL*_Bc_*-his with and without induction*.*** The MSfragger PSMs search summary for the proteome analysis of Δ*amrAB*Δ*pglL* and Δ*amrAB*Δ*pglL* containing chromosomally integrated CTX-CymR_GV_-PglL*_Bc_*-his with and without induction are provided. For each modification type identified within the proteome the total number of PSMs assigned are tabulated.

**Supplementary Table 11. DDA peptide and protein identification tables of *B. cenocepacia* K56-2 Δ*amrAB*Δ*pglL* and Δ*amrAB*Δ*pglL* containing chromosomally integrated CTX-CymR_GV_-PglL*_Bc_*-his with and without induction*.*** MSfragger search results for the protein analysis of four biological replicates of strains *B. cenocepacia* K56-2 Δ*amrAB*Δ*pglL* and Δ*amrAB*Δ*pglL* containing chromosomally integrated CTX-CymR_GV_-PglL*_Bc_*-his without and with induction with 100 μM cumate. For each peptide and protein, the identification information (including modification assignments) and quantitative information including ion intensities as well as if the assignment was based on matching or by MS/MS identifications across the biological replicates are provided.

**
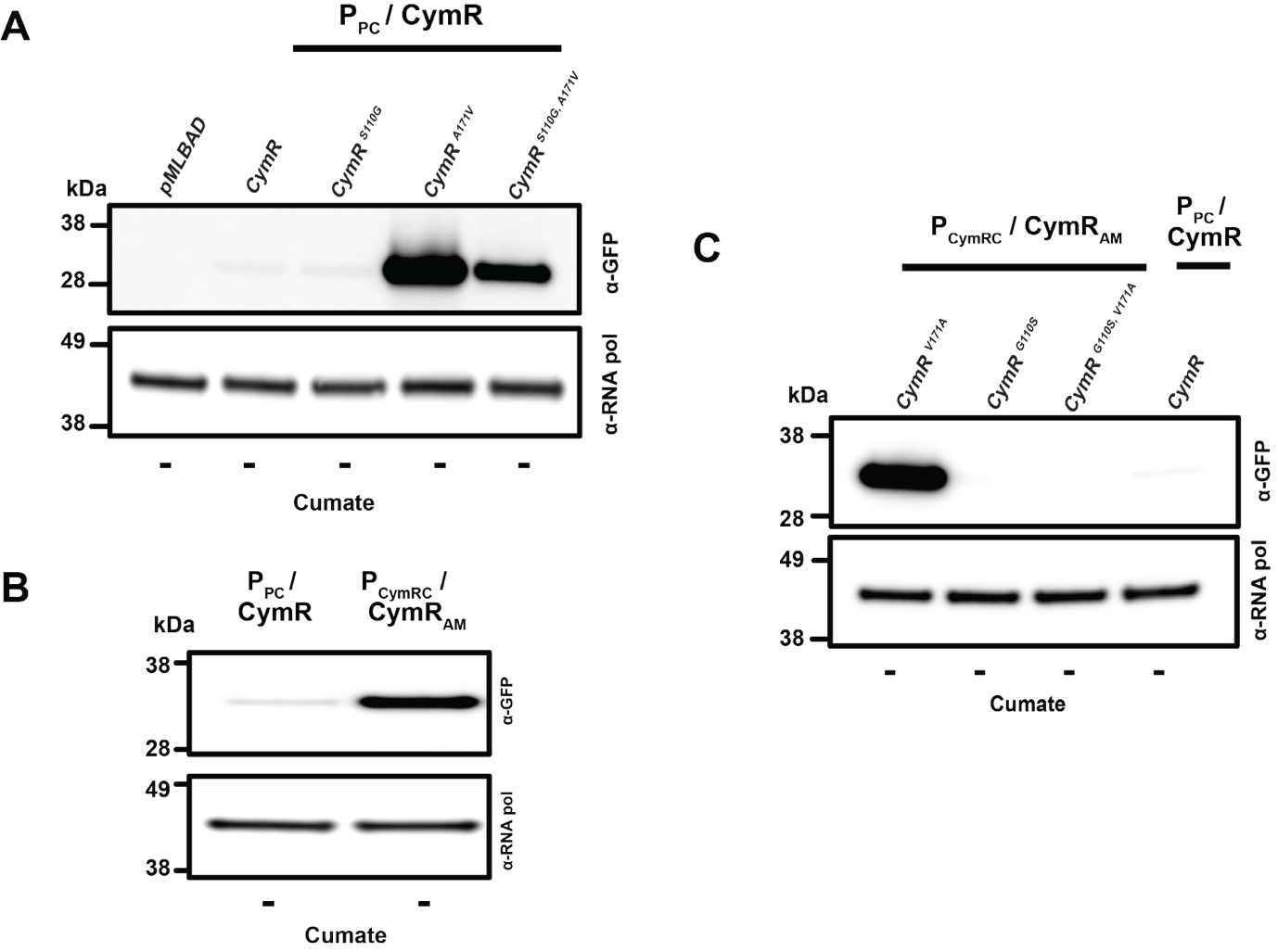
**

**Supplementary Figure 1: Western analysis of basal sfGFP expression of Cumate Circuit variants within *E. coli*. A)** Amino acid substitution of CymR within the P_PC_/CymR cumate circuit results in an increase in basal expression of sfGFP. **B)** Comparison of basal expression between P_pc_/CymR and codon optimized P_CymRC_/CymR_Am_ cumate switch demonstrate the identical increase in expression observed within *B. cenocepacia*. **C)** Amino acid substitution of CymR_AM_ within the P_CymRC_/CymR_AM_ cumate circuit results in an increase in basal expression for V171A.


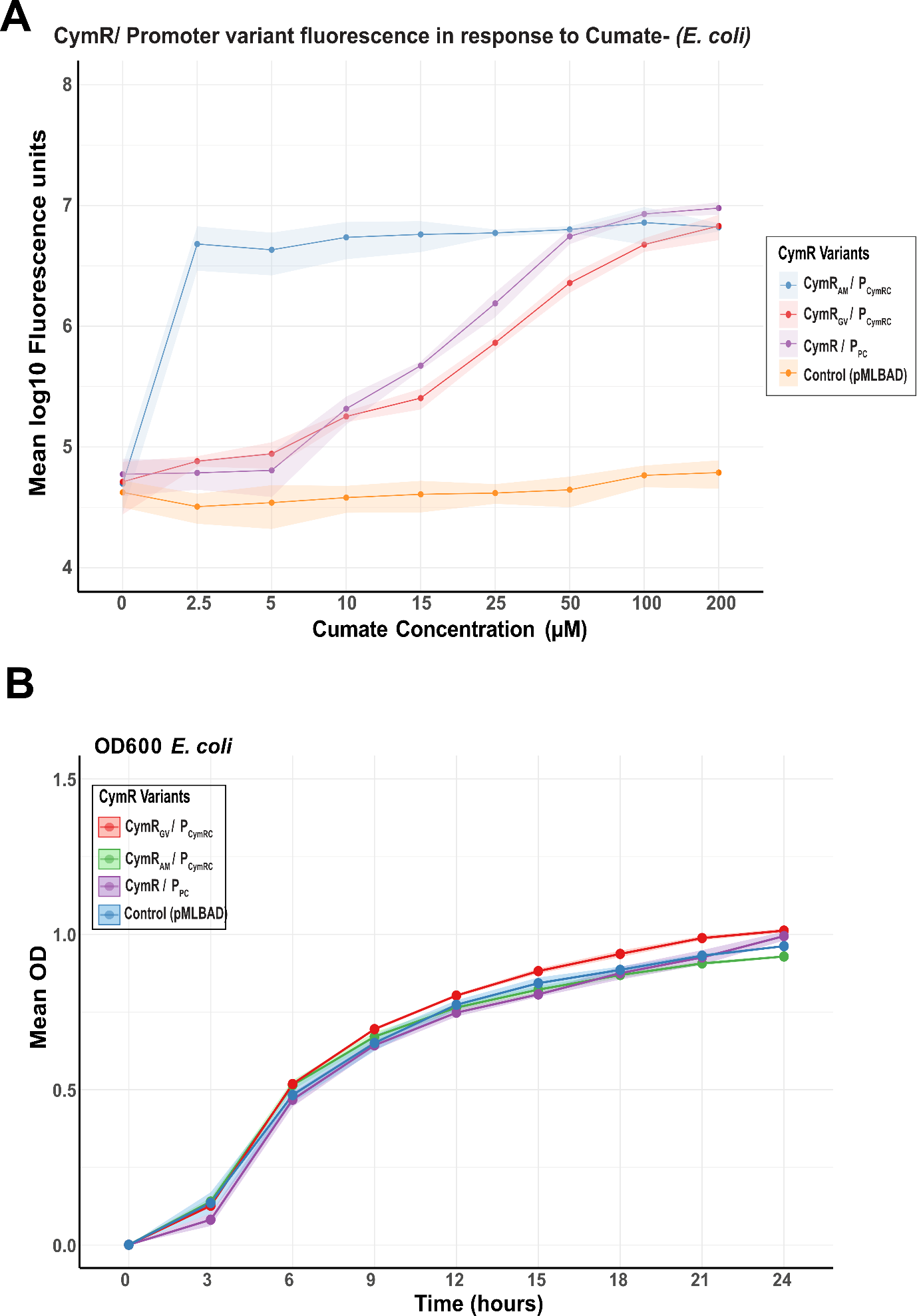


**Supplementary Figure 2: sfGFP fluorescence in response to varying levels of cumate and growth kinetics for cumate variants within *E. coli*. A)** Fluorescence in response to varying levels of cumate induction within *E. coli* following 24 hours of induction. **B)** OD_600_ growth analysis reveals identical growth kinetics of plasmids containing different cumate circuits.

**
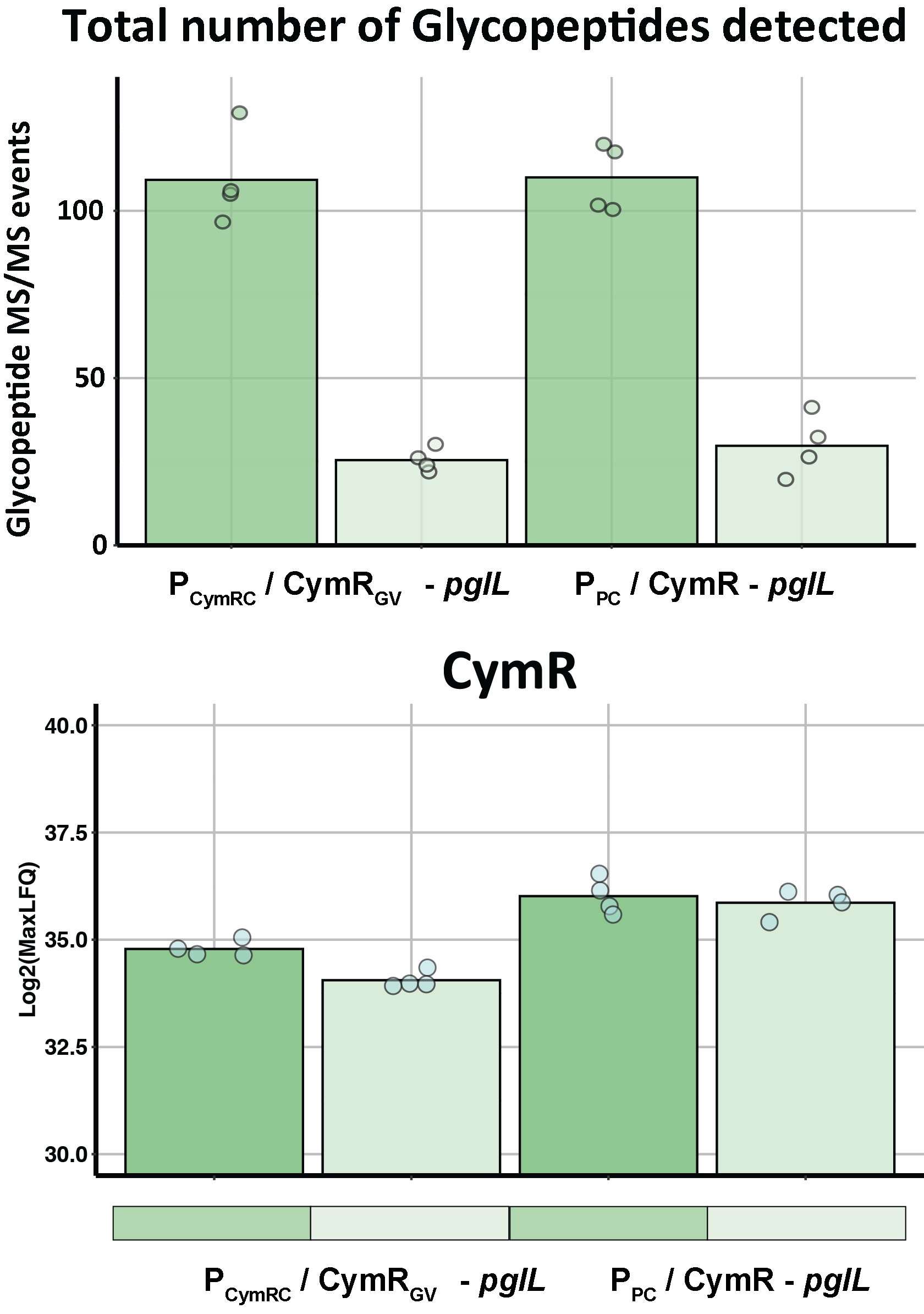
**

**Supplementary Figure 3: Quantification of glycopeptides and CymR levels.** whole cell proteomic analysis of *B. cenocepacia* Δ*pgl*L BCAL1086-His containing either the vector pCumate^GV^-PglL*_Bc_*-his (P_CymRC_/CymR_GV_) or pCumate-PglL*_Bc_*-his (P_Cym_/CymR) with and without induction with 100 uM cumate (n = 4).


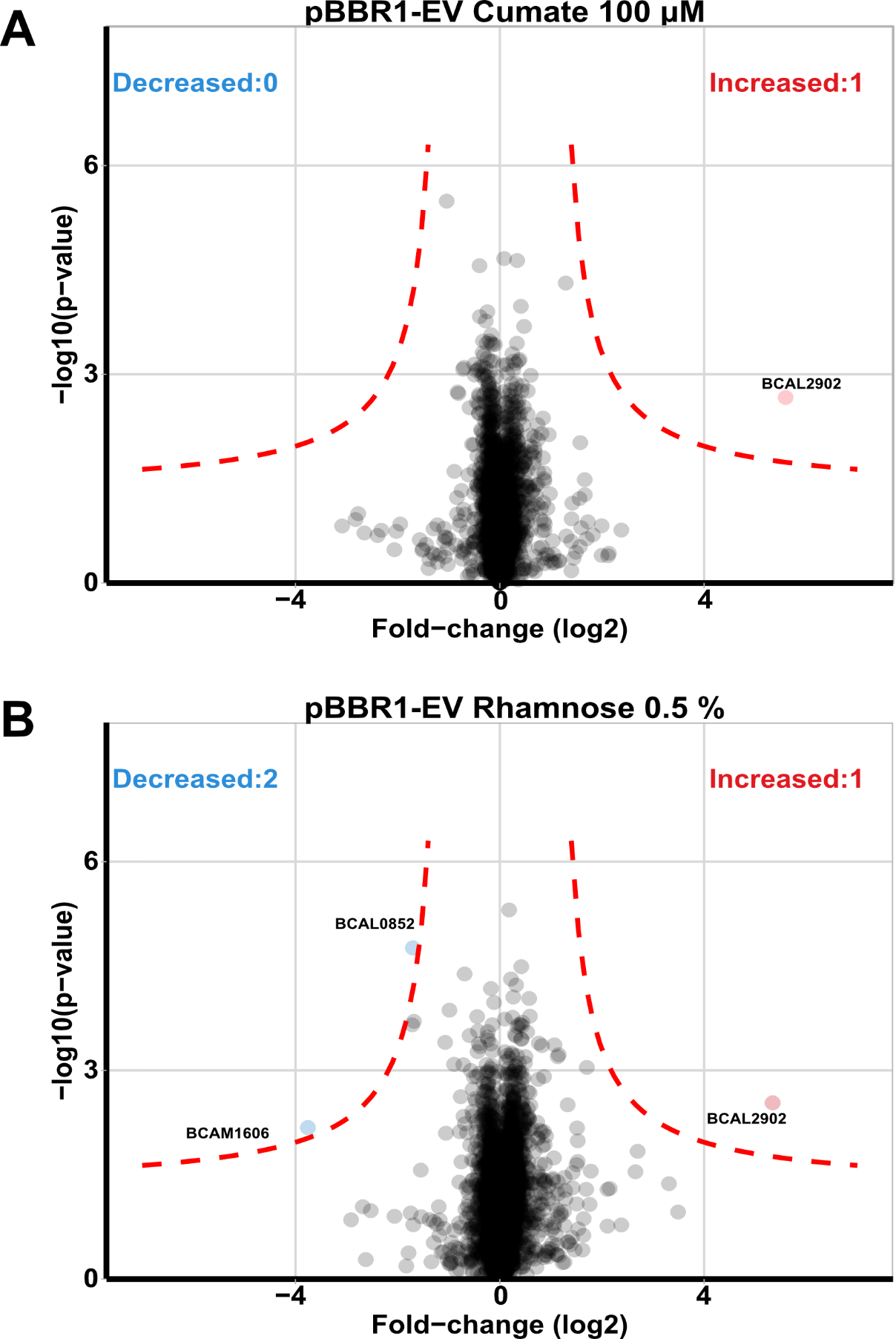


**Supplementary Figure 4: Proteomic analysis of cumate and rhamnose induction in *B. cenocepacia* K56-2 containing pBBR1-EV.** A–B) Volcano plots showing differential protein abundance (log₂ fold-change vs. -log₁₀-adjusted p-value) between induced (100 μM cumate or 0.5% L-rhamnose) for pBBR1-EV.

**
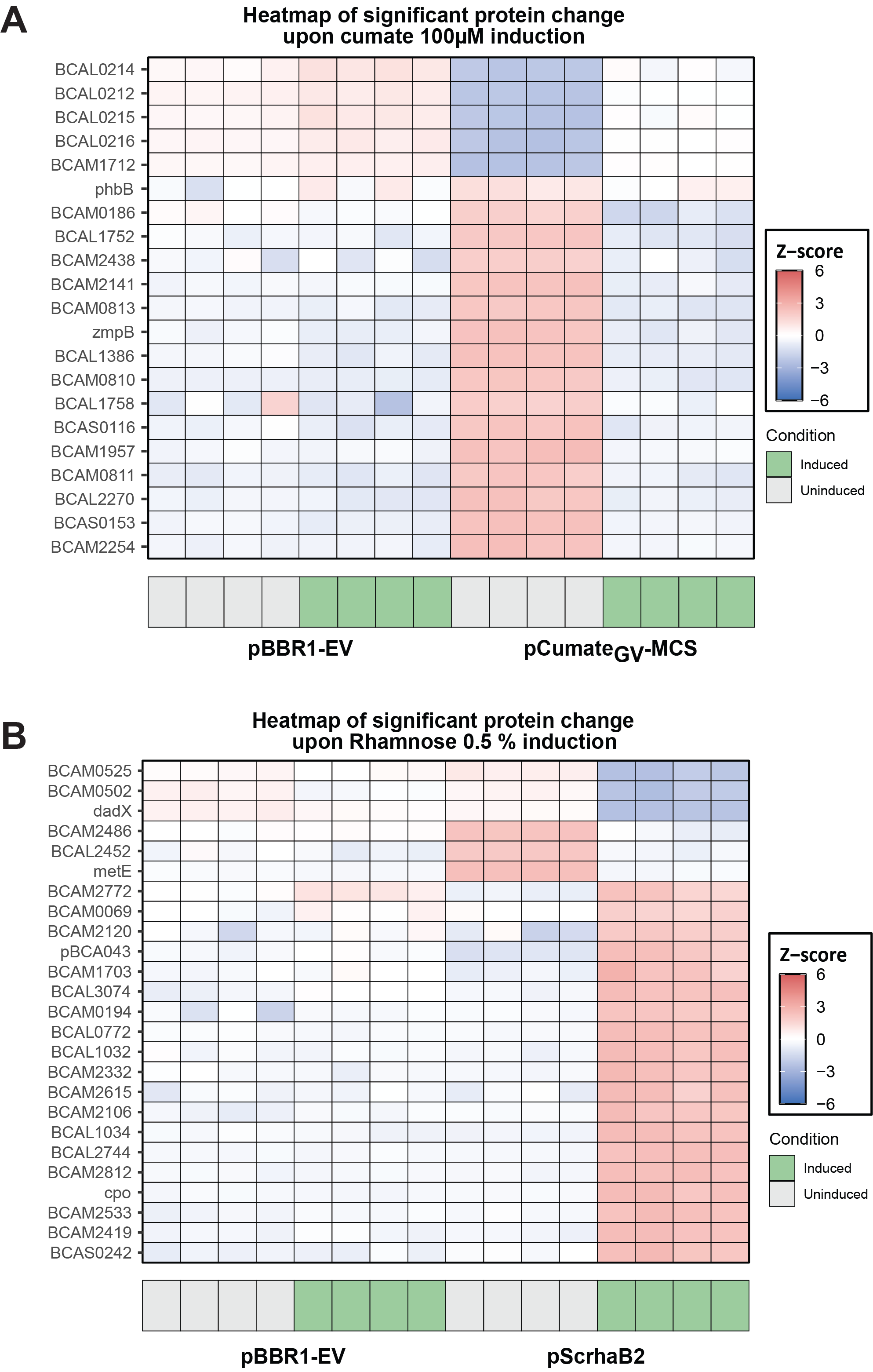
**

**Supplementary Figure 5: Proteomic analysis of cumate and rhamnose induction in *B. cenocepacia* K56-2 containing A)** pCumate_GV_-MCS vs pBBR1-EV in response to cumate **B)** pScrhaB2 vs pBBR1-EV in response to rhamnose.


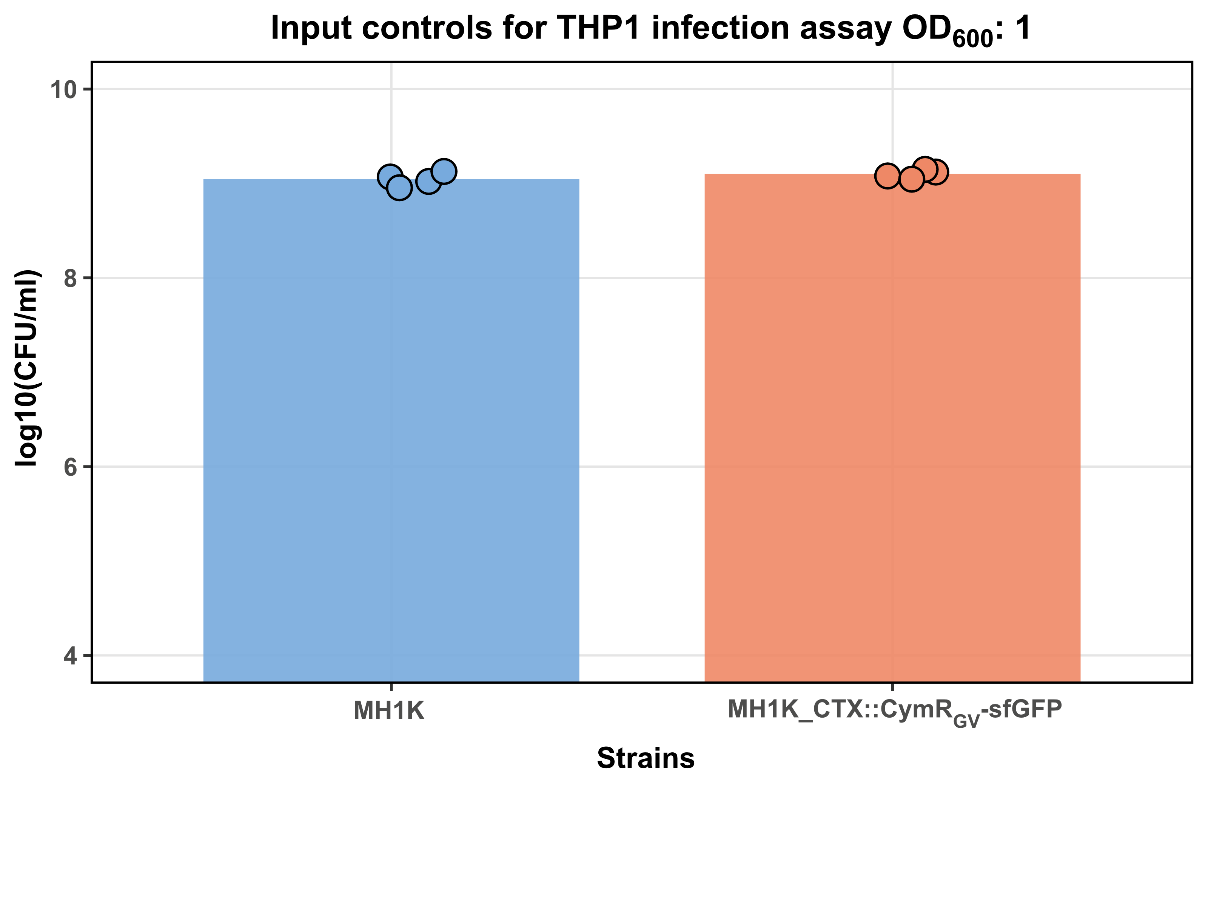


**Supplementary Figure 6:** Input quantification of bacterial inoculum used to infect THP-1 macrophages normalized to OD_600_ 1. CFUs were enumerated for *B. cenocepacia* K56-2 MH1K and MH1K CTX::CymR_GV_-sfGFP strains prior to host cell infection. Data represent four independent biological replicates.


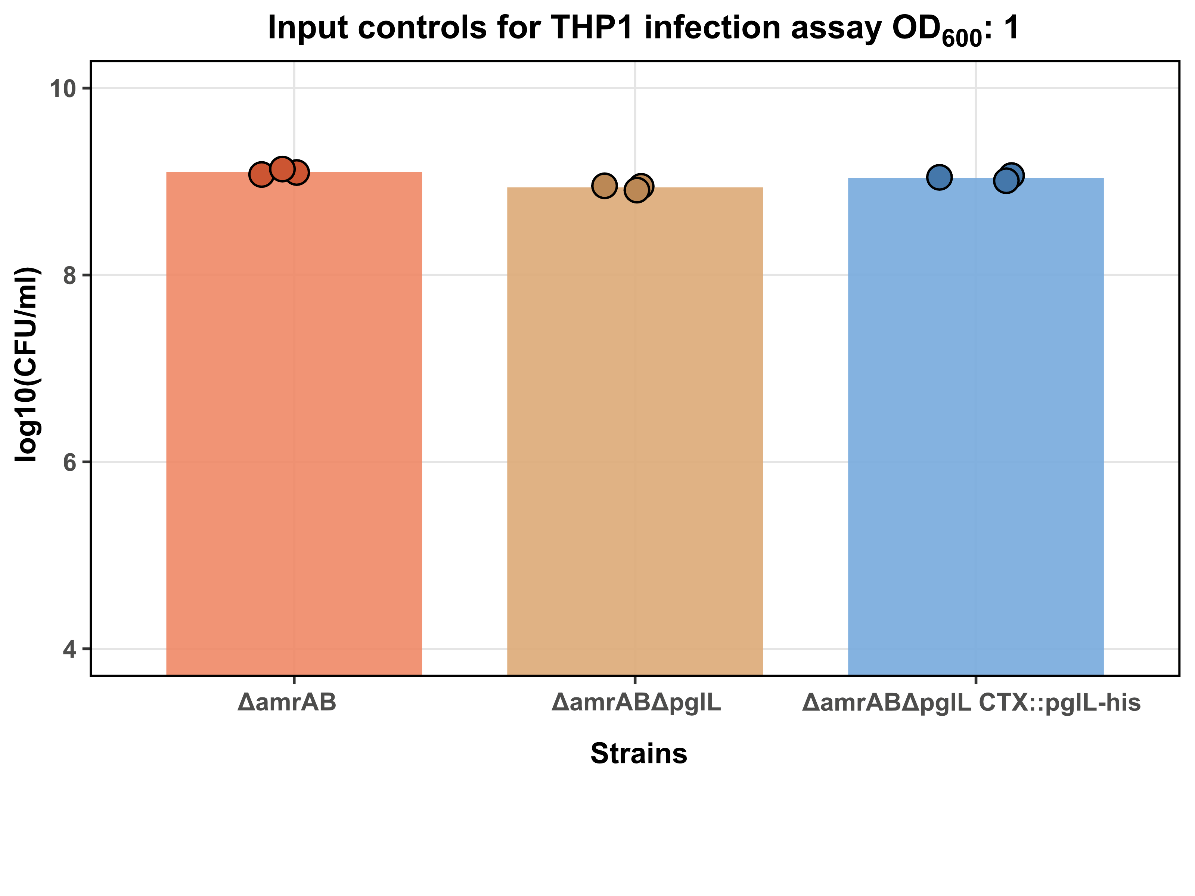


**Supplementary Figure 7:** Input quantification of bacterial inoculums used to infect THP-1 macrophages normalized to OD_600_ 1. CFUs were enumerated for *B. cenocepacia* K56-2 Δ*amr*AB, Δ*amr*ABΔpglL, and Δ*amr*ABΔpglL CTX::CymR_GV_-pglL-His strains prior to infections. Data represents three independent biological replicates.


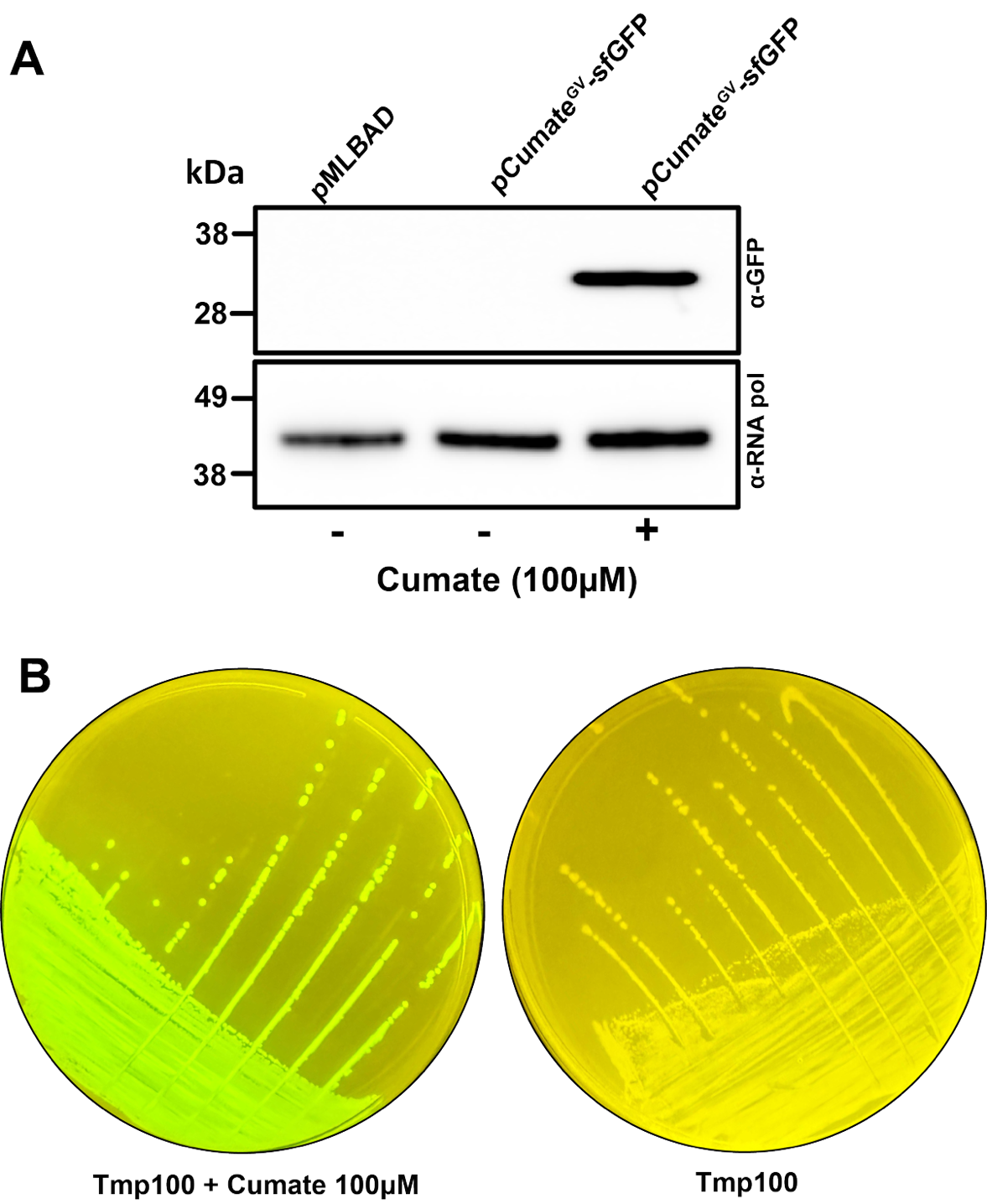


**Supplementary Figure 8: Functionality of pCumate^GV^-sfGFP with *B. thailandensis*. A)** Western blot of sfGFP expression within *B. thailandesis* shows cumate dependent induction. **B)** *B. thailandesis* plates containing cumate allow the induction of sfGFP, leading to visible fluorescence under blue light.


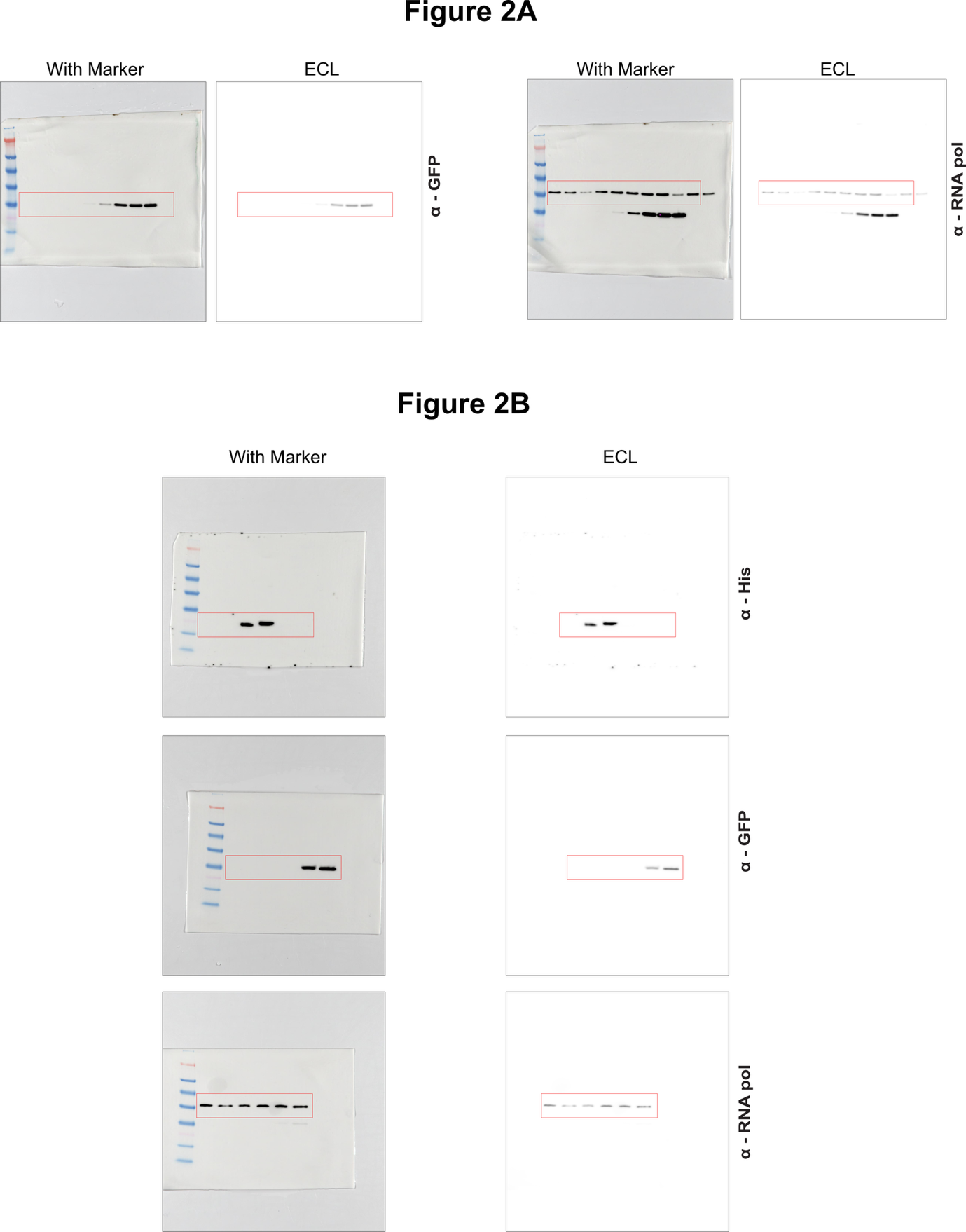


**Supplementary Figure 9. Uncropped Western blotting images.** The uncropped ECL and membrane images for western shown in Figure 2 with the manuscript are provided.


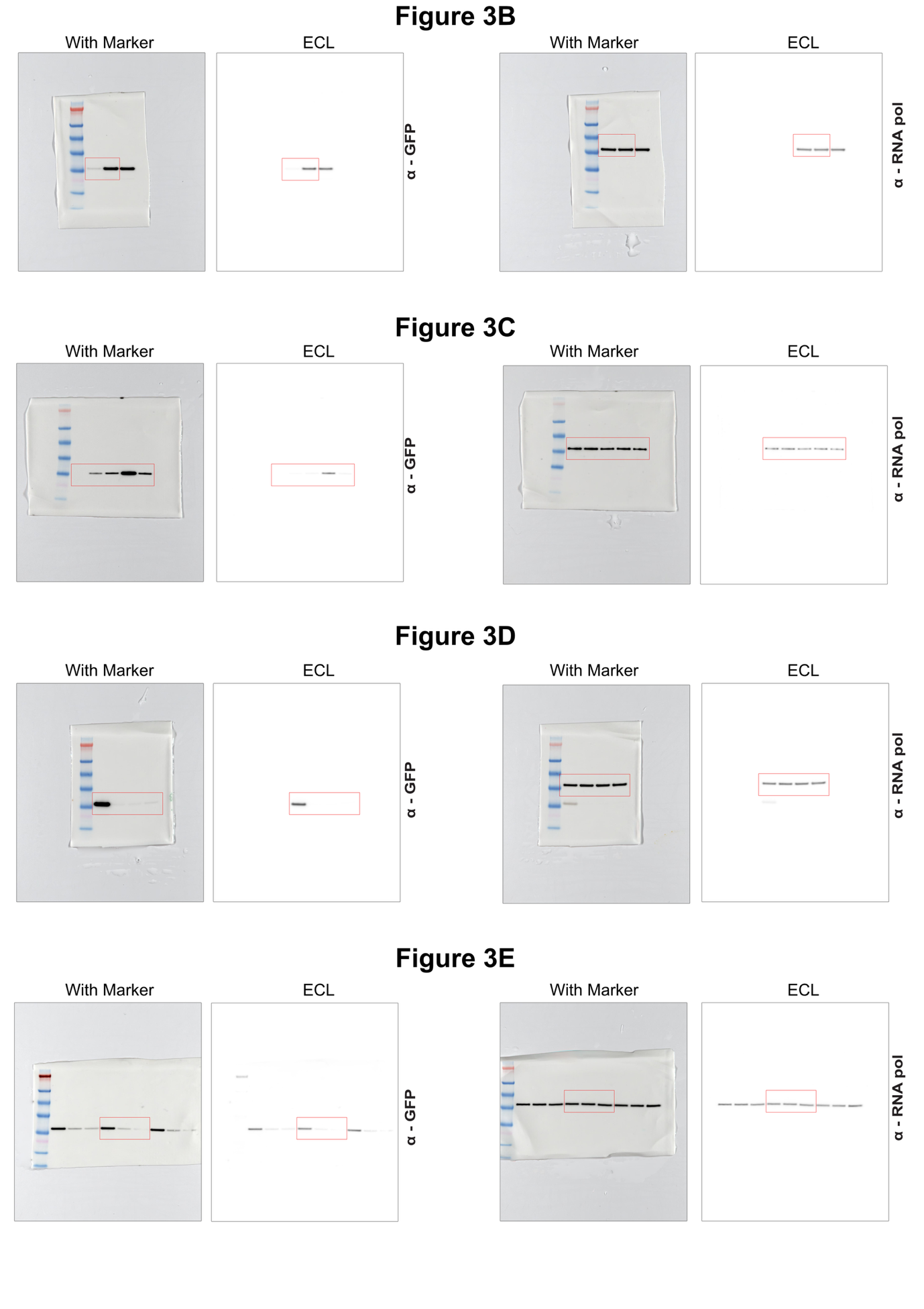


**Supplementary Figure 10. Uncropped Western blotting images.** The uncropped ECL and membrane images for western shown in Figure 3 with the manuscript are provided.

**
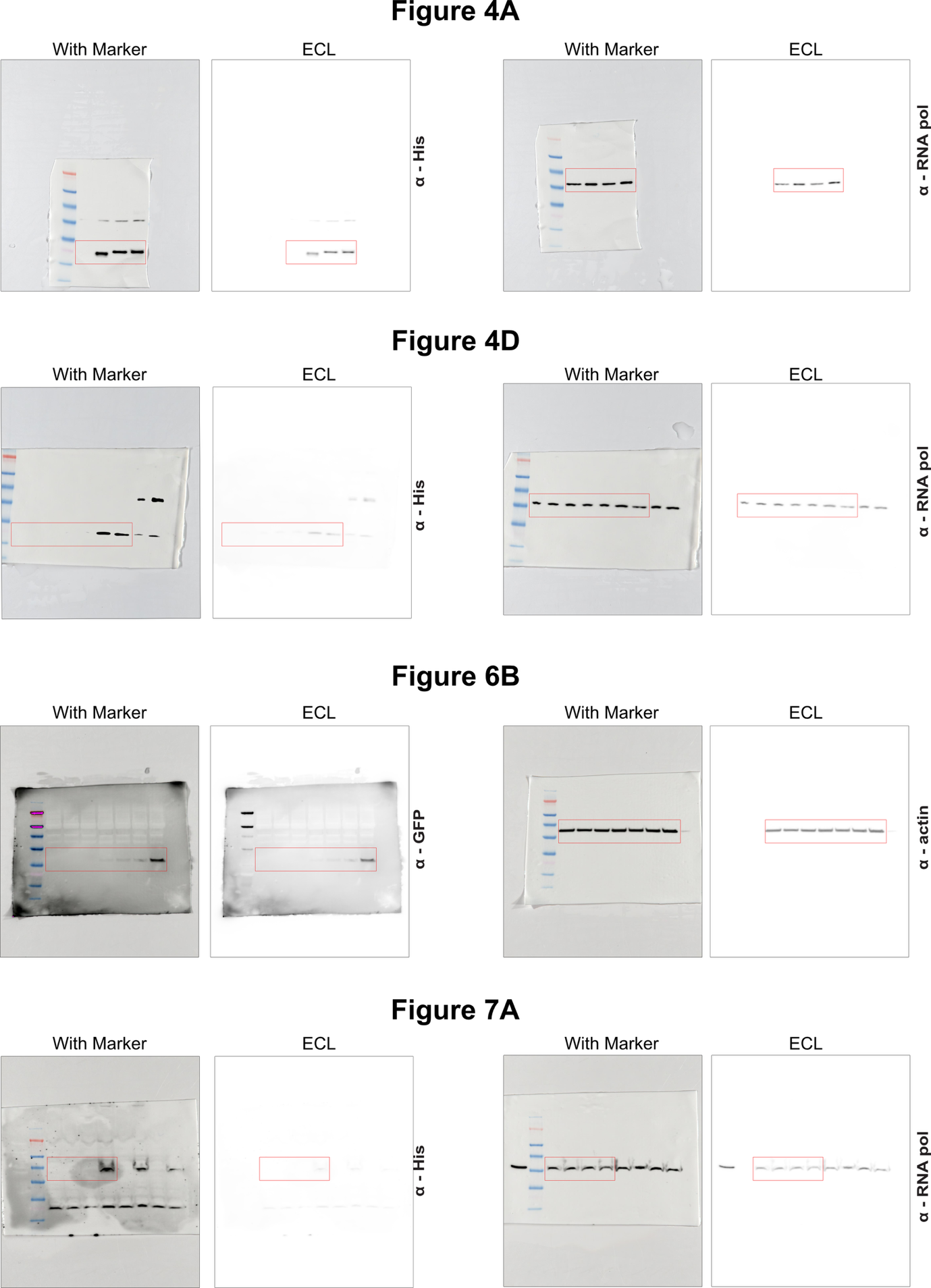
**

**Supplementary Figure 11. Uncropped Western blotting images.** The uncropped ECL and membrane images for western shown in Figures 4, 6 and 7 with the manuscript are provided.

1. Darling, P., et al., *Siderophore production by cystic fibrosis isolates of Burkholderia cepacia.* Infect Immun, 1998. **66**(2): p. 874-7.

2. Oppy, C.C., et al., *Loss of O-Linked Protein Glycosylation in Burkholderia cenocepacia Impairs Biofilm Formation and Siderophore Activity and Alters Transcriptional Regulators.* mSphere, 2019. **4**(6).

3. Hamad, M.A., A.M. Skeldon, and M.A. Valvano, *Construction of aminoglycoside-sensitive Burkholderia cenocepacia strains for use in studies of intracellular bacteria with the gentamicin protection assay.* Appl Environ Microbiol, 2010. **76**(10): p. 3170-6.

4. Brett, P.J., D. DeShazer, and D.E. Woods, *Note: Burkholderia thailandensis sp. nov., a Burkholderia pseudomallei-like species.* International Journal of Systematic and Evolutionary Microbiology, 1998. **48**(1): p. 317-320.

5. Lefebre, M.D. and M.A. Valvano, *Construction and evaluation of plasmid vectors optimized for constitutive and regulated gene expression in Burkholderia cepacia complex isolates.* Appl Environ Microbiol, 2002. **68**(12): p. 5956-64.

6. Hamad, M.A., et al., *Aminoarabinose is essential for lipopolysaccharide export and intrinsic antimicrobial peptide resistance in Burkholderia cenocepacia(dagger).* Mol Microbiol, 2012. **85**(5): p. 962-74.

7. Figurski, D.H. and D.R. Helinski, *Replication of an origin-containing derivative of plasmid RK2 dependent on a plasmid function provided in trans.* Proc Natl Acad Sci U S A, 1979. **76**(4): p. 1648-52.

8. Barrett, A.R., et al., *Genetic tools for allelic replacement in Burkholderia species.* Appl Environ Microbiol, 2008. **74**(14): p. 4498-508.

9. Cardona, S.T. and M.A. Valvano, *An expression vector containing a rhamnose-inducible promoter provides tightly regulated gene expression in Burkholderia cenocepacia.* Plasmid, 2005. **54**(3): p. 219-28.

10. Schuster, L.A. and C.R. Reisch, *A plasmid toolbox for controlled gene expression across the Proteobacteria.* Nucleic Acids Res, 2021. **49**(12): p. 7189-7202.

11. Hogan, A.M., et al., *A Broad-Host-Range CRISPRi Toolkit for Silencing Gene Expression in Burkholderia.* ACS Synth Biol, 2019. **8**(10): p. 2372-2384.

**References**
